# Explicit solvation thermodynamics in ionic solution: extending grid inhomogeneous solvation theory to solvation free energy of salt–water mixtures

**DOI:** 10.1007/s10822-021-00429-y

**Published:** 2022-01-15

**Authors:** Franz Waibl, Johannes Kraml, Monica L. Fernández-Quintero, Johannes R. Loeffler, Klaus R. Liedl

**Affiliations:** Department of General, Inorganic, and Theoretical Chemistry, University of Innsbruck, Innrain 80/82, 6020 Innsbruck, Austria

**Keywords:** Grid inhomogeneous solvation theory, Free energy of solvation, Salt, Mixture, Solvation, Salting-out

## Abstract

Hydration thermodynamics play a fundamental role in fields ranging from the pharmaceutical industry to environmental research. Numerous methods exist to predict solvation thermodynamics of compounds ranging from small molecules to large biomolecules. Arguably the most precise methods are those based on molecular dynamics (MD) simulations in explicit solvent. One theory that has seen increased use is inhomogeneous solvation theory (IST). However, while many applications require accurate description of salt–water mixtures, no implementation of IST is currently able to estimate solvation properties involving more than one solvent species. Here, we present an extension to grid inhomogeneous solvation theory (GIST) that can take salt contributions into account. At the example of carbazole in 1 M NaCl solution, we compute the solvation energy as well as first and second order entropies. While the effect of the first order ion entropy is small, both the water–water and water–ion entropies contribute strongly. We show that the water–ion entropies are efficiently approximated using the Kirkwood superposition approximation. However, this approach cannot be applied to the water–water entropy. Furthermore, we test the quantitative validity of our method by computing salting-out coefficients and comparing them to experimental data. We find a good correlation to experimental salting-out constants, while the absolute values are overpredicted due to the approximate second order entropy. Since ions are frequently used in MD, either to neutralize the system or as a part of the investigated process, our method greatly extends the applicability of GIST. The use-cases range from biopharmaceuticals, where many assays require high salt concentrations, to environmental research, where solubility in sea water is important to model the fate of organic substances.

**Supplementary Information:**

The online version contains supplementary material available at 10.1007/s10822-021-00429-y.

## Introduction

“Water, water, every where, ne any drop to drink”, the ancient mariner cries out in Samuel Coleridge’s poem [1–5]. And, in fact, water is one of the most ubiquitous molecules on earth. 71% of earth’s surface are covered by water, and most biochemical reactions occur in aqueous solution [6, 7]. However, most water is not pure, but part of a solution. Sea water is undrinkable due to its high salt concentration, and the human serum contains a multitude of organic and inorganic solutes. Furthermore, salt strongly impacts the structure and function of biomolecules, such as proteins [8] or DNA [9, 10].

There has been large scientific interest in predicting the interactions of water with dissolved substances [9]. In the field of environmental research, solubility of organic compounds in sea water determines their evaporation as well as their bioaccumulation [11]. In pharmaceutical research, hydration properties of large molecules are fundamental to the understanding of aggregation [12], solubility [13], viscosity, and protein expression.

Many biochemically relevant processes depend not only on the solute-water interaction, but also on the salt concentration. For example, ions influence the melting temperature [14] and conformations [15] of DNA, as well as the stability of proteins in solution, an effect that is described by the Hofmeister series [16]. They also affect many biochemical assays, such as Hydrophobic Interaction Chromatography (HIC) [17], which is routinely applied to detect surface hydrophobicity of biopharmaceuticals [18], and utilizes a salt gradient to control elution. It has been shown that results from Affinity-Capture Self-Interaction Nanoparticle Spectroscopy (AC-SINS) [19] correlate with HIC experiments only when sufficient salt concentrations are used. This is expected since long-range electrostatic interactions are shielded, while hydration of hydrophobic side chains as well as the protein backbone is disfavored by the salting-out effect [20].

To improve the understanding of biomolecular solvation, it is crucial to develop computational methods that can describe the effect of salts on hydration properties. For in-depth analysis of solvation effects, it is desirable that those methods provide separate contributions of energy and entropy to the free energy of solvation, as well as a spatial resolution of the computed properties [21].

In this work, we extend the well-known GIST [22] method, which computes spatially resolved contributions to the free energy of solvation based on molecular dynamics (MD) simulations, to take salt effects into account. In the following, we will give a short introduction to the salting-out effect, before reviewing existing computational approaches to describe molecular solvation properties.

### The salting-out effect

Setschenow showed a linear correlation between the logarithm of the solubility *S* of a compound and the salt concentration in the solvent [23], called the Setschenow relation.

1 $$\mathrm{log}\frac{S}{{S}_{0}}={K}_{S}\times {c}_{\text{salt}}$$

here, *K*_S_ is the salting-out constant and depends on the nature of the solute as well as the solvent, and *c* is the salt concentration. *S*_0_ is the solubility in of the compound in pure water.

Some trends have been observed regarding *K*_S_. Generally, small and highly charged ions tend to reduce the solubility of hydrophobic solutes, while larger ions are less strongly hydrated and tend to be more favoring to hydration of hydrophobic compounds [24, 25]. Charged and polar compounds tend to have a weaker salting-out effect, or even a salting-in effect (negative *K*_S_) [24]. There is also an approximately linear dependence between the size of a solute and its *K*_S_.

Multiple methods, including scaled-particle theory [26], Kirkwood–Buff theory [27], and test particle insertion [28, 29], have been used to describe the salting-out effect of nonpolar gases.

Here, we use the salting-out effect as a test system for our generalized GIST approach, since it can be directly linked to the free energy of solvation of a compound in dependence of the salt concentration. For more details, we refer to the Supporting Information (SI).

### Computational approaches to solvation

In the field of large biomolecules, most methods dispense with any physical description of the solvent, instead treating quantities like solubility or hydrophobicity as intrinsic to the solute. These tools have proven useful at highly complex tasks such as reducing the aggregation propensity of antibodies [30, 31]. However, they do not provide a physical description of the solvent and are therefore inadequate to describe the effect of the solvent composition and salt concentration.

The solubility of organic substances is frequently described using implicit solvent models, that treat both the water and the dissolved ions as a homogeneous continuum. Those methods provide a simple, yet effective approach to model different salt concentrations. For example, the statistical associating fluid theory (SAFT) [32], as well as the conductor-like screening model (COSMO) in combination with quantum mechanics [33, 34] have been used successfully to predict the effect of salt concentration on solubility. However, it has been shown that the accuracy of implicit solvent models is limited compared to explicit methods based on MDs simulation [35, 36].

Multiple theories have been developed to describe solvation of hydrophobic molecules, many of which are able to incorporate salts as part of the solvent. Notable examples include scaled particle theory [37], Kirkwood–Buff theory [27, 38], as well as approaches based in information theory [39] and test particle insertion [27, 40]. Typically, those methods focus on hydration of purely hydrophobic compounds, though they might be complemented by additional energy terms [41].

Thermodynamic integration (TI) [42–44] is a method that calculates free energy differences based on alchemical transformations, such as replacement of a chemical group. It can accurately compute absolute hydration free energies by slowly removing the solute from the simulation box. This is a very general method and has been successfully applied to solvents containing ions [45]. However, care has to be taken when the solute molecule carries a net charge [46–48]. Furthermore, the applicability of TI is limited to rather small molecules.

Methods that explicitly model the statistical mechanics of the solvent can be classified broadly into those based on the Ornstein–Zernike (OZ) equation [49] and those based on inhomogeneous solvation theory (IST) [50]. Methods based on the OZ equation, such as 3D-RISM [51, 52], compute the distribution of particle densities from a molecular mechanics force field in combination with the pair distribution function, and can treat both pure solvents and salt–water mixtures [53]. It has also been shown that 3D-RISM can be used to compute salting-out coefficients [54].

In contrast, methods based on IST [50, 55] use an ensemble of solvent conformations, typically obtained from MD simulation, to estimate the partition sum of the solvent molecules in the proximity of the solute. A discretized implementation of IST, called grid inhomogeneous solvation theory (GIST), has been applied to a wide range of biomolecular systems, such as solvation thermodynamics of the cucurbit[7]uril receptor [22], unfavorable hydration sites on the surface of proteins in the context of protein–drug interactions, binding characteristics of serine proteases [56], or hydrophobicity of antibodies [57]. Recent developments include improved performance due to GPU-acceleration [56] or PME-based energy calculation [58], as well as an extension to solvents other than water [59].

Further methods based on IST include WaterMap [60], SSTMap [61] and STOW [62]. However, none of the IST-based methods has been extended to account for the presence of salt in the solvent. This is problematic not only due to the large number of biochemical processes that are affected by the salt content of the solvent, but also because MDs simulations of charged systems are commonly performed using counter ions. Prior GIST studies either did not use counter ions [57], or restrained them as part of the solute [63].

Here, we present an extension of the GIST algorithm that explicitly treats ions as part of the solvent and test its validity on the solvation free energy of carbazole in 1 M NaCl solution. We compute first and second order entropies and test the viability of the Kirkwood superposition approximation (KSA) for a simplified computation of second order entropies. Furthermore, we compute a set of salting-out coefficients from previous work by Endo et al. [34]. This allows us to estimate the accuracy of our method and to show opportunities for prospective improvements of the entropy estimation in GIST.

## Theory

### GIST

Here, we shortly review the theory behind existing GIST implementations. The extension to multiple solvents, as well as the second order entropy estimate, will be discussed below.

GIST is a method devised by Nguyen et al. [22, 64]. It uses IST [50] to compute thermodynamic quantities of water in the vicinity of a solute, but replaces the spatial integrals by discrete sums over voxels in a 3-dimensional grid.

The input of a GIST calculation is the trajectory of an MD simulation of the solvent molecules surrounding a restraint solute.

The free energy of solvation Δ*A*_solv_ is split into separate contributions for energy and entropy.

2 $$\Delta{A}_{solv}=\Delta{E}_{solv}-T\Delta{S}_{solv}$$

here, *E*_solv_ denotes the energy contribution to solvation, while *S*_solv_ is the entropy contribution. T is the system temperature, and the Δ denotes that quantities are calculated relative to their respective bulk value.

The energy is further split into contributions for the solute–solvent (*E*^uv^) and solvent–solvent (*E*^vv^) interactions.

3 $$\Delta {E}_{solv}=\Delta {E}_{solv}^{uv}+\Delta {E}_{solv}^{vv}$$

Both energy terms can be readily computed from the force field. For *E*^uv^, interaction energies are calculated between each solvent molecule and the solute, and assigned to the grid voxel that currently holds the solvent molecule. The same is done in every frame.

Similarly, *E*^vv^ is calculated from the interaction energies between all pairs of solvent molecules. Half of the energy contribution is assigned to each of the two molecules, to avoid double counting.

The solute–solvent contribution goes to zero with increasing distance from the solute, i.e., *E*^uv^ is zero in the bulk. In contrast, *E*^vv^ tends to a constant value (*E*^vv^_bulk_) in the bulk. This value must be subtracted to obtain the energy change of the solvent upon addition of the solute.

4 $$\Delta {E}_{solv}^{vv}={E}_{solv}^{vv}-{E}_{bulk}^{vv}$$

The entropy contribution can be calculated in two different ways. First, it can be further separated into contributions for the translational (*S*^trans^) and the rotational (*S*^orient^) entropy.

5 $$\Delta {S}_{solv}\cong \Delta {S}_{\text{solv}}^{\text{trans}}+\Delta {S}_{\text{solv}}^{\text{orient}}$$

Alternatively, a single entropy estimate (*S*^six^) can be computed from all six degrees of freedom of the solvent molecule.

6 $$\Delta{S}_{\text{solv}}\cong \Delta{S}_{\text{solv}}^{\text{six}}$$

In practice, all three entropy quantities (trans, orient, and six) are calculated using a nearest-neighbor estimate. For each voxel *k*:

7 $${S}_{k}^{\text{trans}}=R\left(\gamma +\frac{1}{{N}_{k}}\sum_{i=1}^{{N}_{k}}{\text{ln}}\frac{{N}_{f}{\rho }^{0}4\pi \cdot {d}_{trans}^{3}}{3}\right)$$

8 $${S}_{k}^{\text{orient}}=R\left(\gamma +\frac{1}{{N}_{k}}\sum_{i=1}^{{N}_{k}}{\text{ln}}\frac{{N}_{k}{\left(\Delta {\omega }_{i}\right)}^{3}}{6\pi } \right)$$

9 $${S}_{k}^{\text{six}}=R\left(\gamma +\frac{1}{{N}_{k}}{\sum }_{i=1}^{{N}_{k}}{\text{ln}}\frac{{N}_{f}{\rho }^{0}\pi {\left(\Delta {\omega }_{i}^{2}+{d}_{i,trans}^{2}\right)}^{3}}{48}\right)$$

here, R denotes the gas constant, γ is the Euler–Mascheroni constant, which accounts for an asymptotic bias, *N*_*k*_ is the number of solvent molecules that are seen in voxel k throughout the simulation, *N*_*f*_ is the number of frames, *ρ*^0^ is the number density of bulk solvent, *d*_trans_ is the translational distance of a solvent molecule to its nearest neighbor, and Δ*ω* is the rotational distance to its nearest neighbor.

Note that the nearest neighbor distances are calculated between solvent molecules from all frames. Therefore, they tend towards zero in the limit of perfect sampling.

Since this entropy estimate only takes first-order terms—such as the position and orientation of solvent molecules—into account, while omitting higher-order terms such as correlations between the orientation of neighboring solvent molecules, the entropy estimates go to zero in the bulk, at least in the case of perfect sampling. Therefore, no reference values are needed for the entropy in theory. However, limited sampling and inaccuracies in the reference density might lead to a small entropy contribution that is left in the bulk. In previous works, this has sometimes been corrected by subtracting a small reference entropy [58].

### Extension of GIST to mixtures

The aim of this work is to extend GIST to solvation in salt–water mixtures, i.e., solvent mixtures containing a regular solvent (e.g., water), a positively charged ionic species (i.e., Na^+^), and a negatively charged ionic species (i.e., Cl^−^). In the following, we denote those compounds as wat (water), M^+^ (cation) and X^−^ (anion). We note, however, that the same considerations apply to arbitrary mixtures of solvents, and that, in combination with the generalization of GIST to multiple solvents presented in [59], GIST calculations of arbitrary solvent mixtures should indeed become possible.

Throughout this work, we consider the salt to be a part of the solvent rather than the solute. Therefore, the reference state for bulk solvent properties must also be a salt-water mixture.

#### Energy terms

As in the original GIST implementation, the energy terms can be calculated from the employed force field in a straight-forward manner. Since the ions are considered part of the solvent, all ion–solute interactions are added to the solute–solvent energy *E*_uv_ of the voxel currently holding the ion. All water–ion and ion–ion interactions are split by two and added to the solvent–solvent energy *E*_vv_ of the voxels holding the respective molecules, consistently with the solvent–solvent interactions in the original GIST implementation.

Subtracting the appropriate reference energy for the solvent–solvent term, however, is less trivial. We consider a reference state where all solvent molecules have been transferred to the bulk. The average energy attributed to such a system can be written as:

10 $$\langle {E}_{vv,ref}\rangle =\langle {\sum }_{i}^{\text{solvent molecules}}{E}_{i,{\text{bulk}}}\rangle = {\sum }_{i}^{\text{solvent molecules}}\langle {E}_{i,{\text{bulk}}}\rangle$$

here, *E*_*vv*,ref_ denotes the reference energy, which is equal to the energy that the solvent molecules had if they were located in the bulk, *E*_*i,*bulk_ denotes the same energy for a single molecule, and the angle brackets 〈.〉 denote the ensemble average. This method of summation assumes that all energies have already been divided by two to avoid double counting, as discussed in [65].

In the original GIST algorithm, this term is expressed by a constant per-molecule energy term. However, the energy contributions of different molecular species must be treated separately. This leads to the following expression:

11 $${\langle {E}_{vv,{\text{ref}}}\rangle }_{k}={\sum }_{i}^{{N}_{k,{\text{wat}}}}\langle {E}_{\text{wat}}\rangle +{\sum }_{i}^{{N}_{k, {\text{M}}^{+}}}\langle {E}_{{\text{M}}^{+}}\rangle +{\sum }_{i}^{{N}_{k, {\text{X}}^{-}}}\langle {E}_{{\text{X}}^{-}}\rangle ={N}_{k,{\text{wat}}}\times \langle {E}_{\text{wat}}\rangle +{N}_{k, {\text{M}}^{+}}\times \langle {E}_{{\text{M}}^{+}}\rangle +{N}_{k, {\text{X}}^{-}}\times \langle {E}_{{\text{X}}^{-}}\rangle$$

here, *N*_*k,i*_ denotes the number of molecules of type i in voxel k. We treat the expectation value of the energy of each compound in bulk as a constant. We determine those constants by an ordinary least squares (OLS) fit of the solute–solute energy in bulk solution, treating the *N*_*k,i*_ values as independent variables. This fit must be done separately for each solvent composition since different ion concentrations lead to different expectation values of the energy per molecule. To ensure that the solvent is sufficiently bulk-like, we can either perform separate simulations of the solvent without solute or select grid voxels far away from the solute molecule. Throughout this work, the second approach was employed, with a minimum distance of 16 Å from the solute.

We then calculate the reference energy of each voxel k in the vicinity of the solvent by evaluating Eq. 11 using the respective molecule counts in voxel k, as well as the previously determined expectation values of the bulk energy.

#### Entropy terms

Traditionally, GIST only considers first-order entropy terms, omitting all solvent–solvent correlations. This allows us to split the entropy into separate terms for each component of the solvent mixture:

12 $${S}_{k}={S}_{k,{\text{wat}}}+{S}_{k,{\text{M}}^{+}}+{S}_{k,{\text{X}}^{-}}$$

This applies to all three types of entropy terms (translational, orientational, and the six-dimensional entropy). We calculate all terms according to Eqs. 7–9 but use the appropriate number density for each species.

The ions (M^+^ and X^−^) do not have any orientational entropy contribution since they are monoatomic. Therefore, only *S*^trans^ needs to be evaluated for them.

#### Second order entropy

Current GIST implementations do not contain the second-order entropy terms, which describe the change in solvent–solvent structure upon addition of the solute. Recently, it has been shown empirically, in the case of organic molecules in pure water, that a rough estimate of the higher order entropy terms may be obtained by scaling the first order entropy by a constant factor [66].

Since this has been shown only empirically, we cannot assume that this approach can be extended to solvent mixtures. Therefore, we need to estimate ${g}^{inh}$ from MD simulation data. The exact second order entropy in IST is:

13 $$\frac{\Delta {S}^{2nd}}{k}=-\frac{1}{2}{\sum }_{\nu }{\sum }_{{\nu }{^{\prime}}}{\rho }_{\nu }^{\infty }{\rho }_{{\nu }{^{\prime}}}^{\infty }{\int }_{local}d{\varvec{r}} d{{\varvec{r}}}{^{\prime}} {G}_{s\nu }\left({\varvec{r}}\right)\left[{G}_{s{\nu }{^{\prime}}}\left({{\varvec{r}}}{^{\prime}}\right) \left({g}_{\nu ,{\nu }{^{\prime}}}^{inh}\left({\varvec{r}}, {{\varvec{r}}}{^{\prime}}\right)\mathrm{ln}\left({g}_{\nu ,{\nu }{^{\prime}}}^{inh}\left({\varvec{r}}, {{\varvec{r}}}{^{\prime}}\right)\right)-{g}_{\nu ,{\nu }{^{\prime}}}^{inh}\left({\varvec{r}},{{\varvec{r}}}{^{\prime}}\right)+1\right)-\left({g}_{\nu {\nu }{^{\prime}}}^{0}\left({{\varvec{r}},{\varvec{r}}}^{\boldsymbol{^{\prime}}}\right)\mathrm{ln}{g}_{\nu {\nu }{^{\prime}}}^{0}\left({{\varvec{r}},{\varvec{r}}}^{\boldsymbol{^{\prime}}}\right)- {g}_{\nu {\nu }{^{\prime}}}^{0}\left({{\varvec{r}},\boldsymbol{ }{\varvec{r}}}^{\boldsymbol{^{\prime}}}\right)+1\right)\right]$$

here, ν and ν′ denote the solvent species, ${\uprho }^{\infty }$ denotes the solvent density distant to the solute, $G$ is the solute–solvent pair correlation defined with respect to ${\uprho }^{\infty }$. ${g}^{0}$ is the bulk solvent–solvent pair correlation function, which only depends on the relative position and orientation, and ${g}^{inh}$ is the inhomogeneous pair correlation function, which depends on the positions and orientations of both solvent molecules relative to the solute.

In its full form, ${g}^{inh}$ is a 12-dimensional function. In a previous work, Nguyen et al. [67] estimate the translational part of ${g}^{inh}$ on a grid, which is still a 6-dimensional problem and computationally highly challenging. In our case, it is even more difficult to obtain sufficient sampling because the ion density is much lower than that of water. Therefore, we reduce ${g}^{inh}$ to a 1-dimensional radial distribution function (rdf) at each grid voxel, which is still essentially a 4-dimensional function.

Consistently, we also reduce ${g}^{0}$ to a 1D representation ${g}^{0}(d)$. This is exact in the case of spherical solvents.

Denoting the radial distance as $d$, and substituting $\frac{dr}{4{d}^{2}\pi }=\mathrm{d}d$ and ${G}_{s,{\nu }{^{\prime}}}\left({\varvec{r}}, d\right)={\int }_{\left|{r}{^{\prime}}-r\right|=d}{G}_{s,{\nu }{^{\prime}}}\left({{\varvec{r}}}^{\boldsymbol{^{\prime}}}\right) d{{\varvec{r}}}{^{\prime}}/(4{d}^{2}\pi )$, we rewrite Eq. 13 as

14 $$\frac{\Delta {S}^{2nd}}{k}=-\frac{1}{2}{\sum }_{\nu }{\sum }_{{\nu }{^{\prime}}}{\rho }_{\nu }^{\infty }{\rho }_{{\nu }{^{\prime}}}^{\infty }{\int }_{\mathrm{local}}\mathrm{d}{\varvec{r}} \mathrm{d}d 4{d}^{2}\pi {G}_{s\nu }\left({\varvec{r}}\right)\left[{G}_{s{\nu }{^{\prime}}}\left({\varvec{r}}, d\right) \left({g}_{\nu ,{\nu }{^{\prime}}}^{\mathrm{inh}}\left({\varvec{r}}, d\right)\mathrm{ln}\left({g}_{\nu ,{\nu }{^{\prime}}}^{\mathrm{inh}}\left({\varvec{r}}, d\right)\right)-{g}_{\nu ,{\nu }{^{\prime}}}^{\mathrm{inh}}\left({\varvec{r}},d\right)+1\right)-\left({g}_{\nu {\nu }{^{\prime}}}^{0}\left(d\right)\mathrm{ln}{g}_{\nu {\nu }{^{\prime}}}^{0}\left(d\right)- {g}_{\nu {\nu }{^{\prime}}}^{0}\left(d\right)+1\right)\right]$$

Furthermore, we employ the KSA [42] to obtain an estimate of the second order entropy in the same 4D representation. This is the same approach that was used by Lazaridis in his original work on IST [50]. Lately, the KSA has been heavily criticized [68]. Here, we test its validity by comparing between the conditional rdfs and the KSA.

In terms of IST, the KSA assumes that the inhomogeneous pair correlation function between solvent molecules in the vicinity of the solute ${g}^{inh}$ is equal to the bulk pair correlation function ${g}^{0}$.

The KSA simplifies above equation to:

15 $$\frac{\Delta {S}^{2nd,KSA}}{k}= -\frac{1}{2}{\sum }_{\nu }{\sum }_{{\nu }{^{\prime}}}{\rho }_{\nu }^{\infty }{\rho }_{{\nu }{^{\prime}}}^{\infty }{\int }_{\mathrm{local}}\mathrm{d}{\varvec{r}} \mathrm{d}d 4{d}^{2}\pi {G}_{s\nu }\left({\varvec{r}}\right)\left[\left({G}_{s{\nu }{^{\prime}}}\left({\varvec{r}}, d\right)-1\right)\left({g}_{\nu {\nu }{^{\prime}}}^{0}\left(d\right)\mathrm{ln}{g}_{\nu {\nu }{^{\prime}}}^{0}\left(d\right)- {g}_{\nu {\nu }{^{\prime}}}^{0}\left(d\right)+1\right)\right]$$

Since the ions are monoatomic, their pair correlation functions are 1-dimensional. Therefore, the 1D representation is accurate in the case of ion–ion KSA entropies.

We compute the local integral up to a distance of 10 Å, since the pair distribution functions are well converged at this distance.

#### Reference densities

It is well known that the pair distribution function $g$ does not converge to 1 in the canonical ensemble [69], since the central reference particle takes up a certain volume, thereby decreasing the volume that is left to the other particles. IST uses a modified distribution function $G$, that is defined with respect to the density distant to the solute ${\rho }^{\infty }$, rather than the density of an unperturbed bulk solution ${\rho }^{0}$.

Furthermore, IST requires the density of a solvent reference system without the central solute particle. The IST equations contain correction terms that can be applied when the system density does not exactly match the reference density. These terms arise from the enthalpy and entropy of the reference system and are represented as a part of the liberation terms in the final IST equations. The equations may be found in the supporting information or in Lazaridis’ original work [50].

In current GIST implementations, the reference density is needed only for the entropy calculations and must be supplied as an input parameter. When the solvent is pure water, this only needs to be computed once for each combination of water model, temperature, and pressure. With mixtures, the reference density depends on the exact molar fraction of each species, as well as the density of the mixture.

Furthermore, a change in molar composition around the solute will be compensated by more distant regions of the solvent box. For example, small and strongly hydrated species such as F^−^ are strongly disfavored around apolar compounds, which is compensated by a slightly higher density in the “bulk” regions of the solvent box (${\rho }^{\infty }$). The change in bulk density depends on the ratio between the excluded and total volumes and is generally small as long the size of the simulation box is sufficient. Nevertheless, this creates a small, but unphysical dependence of the solvation entropy on the box volume.

Here, we avoid this problem by re-calculating the reference densities ${\rho }^{\infty }$ in each GIST calculation from the bulk-like regions of the solvent box, employing a 12 Å distance-cutoff from the solute. Physically, this implies an infinite system at the bulk solvent composition of the respective solvent box, containing a single solute molecule. This only changes the density by a small amount, because most of the solvent box is bulk-like, due to the small and neutral nature of the molecules in our test set.

We note that this approach implies that the composition of the reference solvent is not known exactly until after the simulation. However, this does not limit the applicability of our method, since it is also trivial to compute the correction terms (the equations are shown in the SI) if the exact solvent composition is required.

#### Implementation details

Typically, the ion concentration is much lower than the water concentration. The current GIST implementations apply a grid-based approach to find the nearest neighbor of each molecule and assume that this neighbor must be in the same or in a neighboring voxel. This is only appropriate if each voxel is visited by multiple molecules during the simulation. However, this is not the case when concentrations much lower than the bulk concentration of water (55.5 M) are used. Therefore, the current implementation of *S*^trans^ is inappropriate to calculate ion entropies. To solve this problem, we created a more general version of the *S*^trans^ calculation using the KD-Tree implementation in either SciPy [70] or pykdtree (https://github.com/storpipfugl/pykdtree) for the nearest-neighbor search. This algorithm works correctly with low concentrations, while yielding identical results as the original implementation for the water entropy. However, it cannot replace the original algorithm due to its lower performance.

For the first order entropy of water, we patched our previously published GPU-accelerated GIST implementation so that the water entropy is calculated correctly in the presence of other solvent molecules. To do so, no algorithmic changes were necessary, but we had to make sure that no M^+^ or X^−^ are counted as water in the entropy calculation. Combining this entropy with the ion entropy from our Python implementation, we obtain the total first order entropy.

For the second order entropy, we discretize ${G}_{s{\nu }{^{\prime}}}({\varvec{r}},d)$ and ${g}_{\nu ,{\nu }{^{\prime}}}^{inh}\left({\varvec{r}}, d\right)$ on a 3-dimensional grid of voxels $k$. Furthermore, we discretize $d$ by radial bins denoted as $l$. We write the discretized quantities as ${G}_{s{\nu }{^{\prime}},kl}$ and ${g}_{\nu {\nu }{^{\prime}},kl}^{inh}$. For every $\nu$ and ${\nu }{^{\prime}}$, we compute the intermolecular distances between all molecules of type $\nu$ that are within the grid, and all molecules of type ${\nu }{^{\prime}}$ in the entire simulation box, using the minimum image convention. We then assign a radial bin $l$ based on the exact distance and increment the respective counter at the respective voxel $k$ holding the first molecule. The resulting histograms are divided by ${N}_{\nu k}{V}_{l}{G}_{s{\nu }{^{\prime}},kl}{\rho }_{\nu }{\rho }_{{\nu }{^{\prime}}}$, to obtain a radial distribution function (rdf). Here, ${N}_{\nu k}$ is the number of molecules $\nu$ found at voxel $k$, ${V}_{l}$ is the bin volume, and ${G}_{{s\nu }{^{\prime}},kl}$ is the density of molecules ${\nu }{^{\prime}}$ in a discretized distance $l$ to voxel $k$.

${G}_{s{\nu }{^{\prime}},kl}$ is computed from a grid representation of the density distribution ${G}_{s{\nu }{^{\prime}}}({{\varvec{r}}}^{\boldsymbol{^{\prime}}})$ by integrating in spherical shells around the center of voxel $k$. To avoid inaccuracies due to the grid spacing, a very fine grid of 0.25 Å spacing is used at first, and the final ${G}_{{s\nu }{^{\prime}},k}(d)$ is re-binned to the desired resolution.

Given the discrete representations of ${g}_{\nu {\nu }{^{\prime}},kl}^{\mathrm{inh}}$, ${g}_{{\nu \nu }{^{\prime}},l}^{0}$ and ${G}_{{s\nu }{^{\prime}},kl}$, the second order entropy can be implemented in a straight-forward manner using Eq. 14. The KSA is used by inserting ${g}_{{\nu \nu }{^{\prime}},l}^{0}$ for ${g}_{\nu {\nu }{^{\prime}},kl}^{inh}$.

Combining the water entropy from the GIST output with the ion entropies calculated in our python implementation, we obtain the total solvation entropy.

The second order entropy and the first order ion entropy are implemented as a series of Python scripts and may be obtained from our group’s GitHub account (https://github.com/liedllab/second-disorder). The updated first order entropy of water is implemented in the newest version of GIGIST.

### Salting-out coefficients

The Salting-Out coefficient *K*_*S*_, also called the Setschenow constant [11, 34], describes the dependence of the solubility of a compound on the salt concentration. It is defined via the Setschenow equation (Eq. 1).

Since solubility is linked to the free energy of solvation Δ*G*_solv_ [71, 72], the Setschenow equation may be formulated as follows:

16 $$\Delta{G}_{\text{solv}}\left({c}_{\text{salt}}\right)=\Delta{G}_{\text{solv}}^{0}+RT{\text{ln}}(10){K}_{S}\times {c}_{\text{salt}}$$

here, T refers to the system temperature in K, and R to the gas constant. Therefore, K_S_ can be obtained from the slope of the free energy of solvation plotted against the salt concentration, divided by RT ln(10). A more detailed derivation of Eq. 16 may be found in the SI.

As described above, we re-calculate the bulk density after the simulations. For consistency, we compute the salt concentration from the GIST reference density. The salt concentration is related to the reference density by Avogadro’s constant N_A_, as well as a conversion factor of 10^27^ from [Å^−3^] to [L^−1^]. Since the concentrations obtained for Na^+^ and Cl^−^ are not numerically equal, the average was used to compute the salting-out coefficient.

## Methods

### Starting structures

We chose 16 rigid molecules out of the dataset by Endo et al. [34]. We excluded all flexible molecules since they require separate GIST calculations for each relevant conformation. Those 16 molecules were used for short GIST calculations (as described below) to compute salting-out coefficients. Furthermore, long simulations of carbazole were run for in-depth analysis of the entropy contributions.

Initial structures for the 16 molecules were obtained from the PubChem database [73]. The structures were optimized using Gaussian 16 [74] at HF/6-31G* level, and charges were obtained via a RESP fit [75]. All other parameters were taken from the Gaff2 force field [76] and assigned using the antechamber and parmchk2 programs in AmberTools19 [77].

All molecules were solvated in cuboid TIP3P [78] water boxes with a minimum distance of 20 Å between the molecule and the walls, using the tleap program from AmberTools. Assuming a constant particle density, solutions at different salt concentrations were produced by replacing a fraction of 1/55*c_salt_ of all water molecules for Na^+^, and the same fraction for Cl^−^, again using tleap. Since this method of setting the salt concentration is not exact, we recompute the exact salt concentration for the calculation of *K*_S_, as described in “Theory” section (“Reference densities” subsection). The Joung-Cheatham ion parameters were used [79].

During all equilibration steps, the solute heavy atoms were restrained using a force constant of 1000 kcal/(mol Å^2^). First, only the hydrogen positions were optimized using 500 steps of steepest descent, followed by 500 conjugate gradient steps. Then, the same was done on all atoms except the solute heavy atoms. The system was then heated from 100 to 300 K within 100 ps, using a time step of 1 fs and a Langevin thermostat [80] with a coupling constant of 2 ps^−1^. Finally, the pressure was equilibrated within 220 ps using a Berendsen barostat [81] with a time constant of 2 ps, at 300 K.

### Molecular dynamics (MD) simulations for GIST

After the equilibration, we performed 200 ns classical MD simulations in the NpT ensemble. The CUDA-accelerated pmemd program in Amber 18 was used. Electrostatic interactions were treated using the PME algorithm [82] with a 8.0 Å cut-off for the real-space contributions. The temperature was set to 300 K using a Langevin thermostat [80] with a collision frequency of 2 ps^−1^, and the pressure was set to 1 bar using the Berendsen barostat [81] with a coupling time of 1 ps. All bond lengths involving hydrogen were constraint using the SHAKE algorithm, [83] allowing for a timestep of 2 fs. Furthermore, all solute heavy atom positions were kept at their initial values using a harmonic restraint of 1000 kcal/(mol Å^2^). Coordinates were stored to disk every 10 ps, resulting in 20,000 frames used in the GIST analysis.

For the second order entropy calculations, we performed a 10 µs MD simulation of water in carbazole using the same starting structure. We stored 10 million frames (1 frame per ps) and kept all other simulation settings equal.

### GIST calculations

After centering the solute coordinates at the origin, GIST analyses were performed using a grid of 81 × 81 × 81 voxels with a spacing of 0.5 Å in every direction. Both the grid and the solute were centered at the coordinate origin. The reference densities were chosen as described in the Theory section and can be found in the SI. A version of the GPU-enabled GIST implementation [22, 50, 59, 84, 85] was used for all contributions except the ion entropy, with minor patches to ensure correct counting of water molecules in the presence of ions. The first order ion entropy and the second order entropies were calculated using our Python implementation (see “Implementation details” in the Theory section).

The free energy of hydration was calculated by integrating the GIST results over all voxels within 6 Å of any heavy atoms of the solute. The six-dimensional entropy estimate was used in favor of the translational and orientational contributions.

### TI calculations

To assess the accuracy of our method in terms of absolute free energy values, we also performed a Thermodynamic Integration (TI) calculation of carbazole in 1.0 M salt solution. The results are shown in “Results and discussion” section, in the subsection titled “Free energy of solvation”.

We used a 2-step TI protocol consisting of an electrostatics part, where the charges of the molecule were removed, and a VdW part, where the already decharged molecule was decoupled from the system using softcore potentials [86, 87].

The 2 steps were started independently. For the electrostatics part, the same equilibrated structure as for the GIST calculations was used. For the VdW part, an extra equilibration step of 25 ns was run before the first lambda window, to account for the loss of charges after the initial equilibration. Soft-core potentials were used with an α parameter of 0.5.

Both steps were composed of 11 equally spaced lambda windows. For the VdW part, we used a simulation time of 25 ns with a time step of 1 fs for each lambda window. Of each window, we discard the first 10 ns as equilibration and use the other 15 ns for analysis. For the electrostatics part, we used 100 ns simulation time at a timestep of 2 fs, discarding the first 40 ns as equilibration.

Furthermore, we ran gas-phase TI calculations as a reference for the electrostatic contribution, using the periodic box of the equilibrated structure without a barostat. This is not required for the VdW part due to the way softcore interactions are handled in Amber.

Free energies profiles were computed separately for the two steps, using the MBAR method as implemented in PyMBAR [88].

### ***K***_***S***_ values

From each GIST or TI run, the salting-out coefficient *K*_S_ and its standard error were computed using Eq. 16 from the slope of Δ*G*_solv_ plotted against the salt concentration *c*.

## Results and discussion

### Energy and first-order ion entropy

**Fig. 1:**
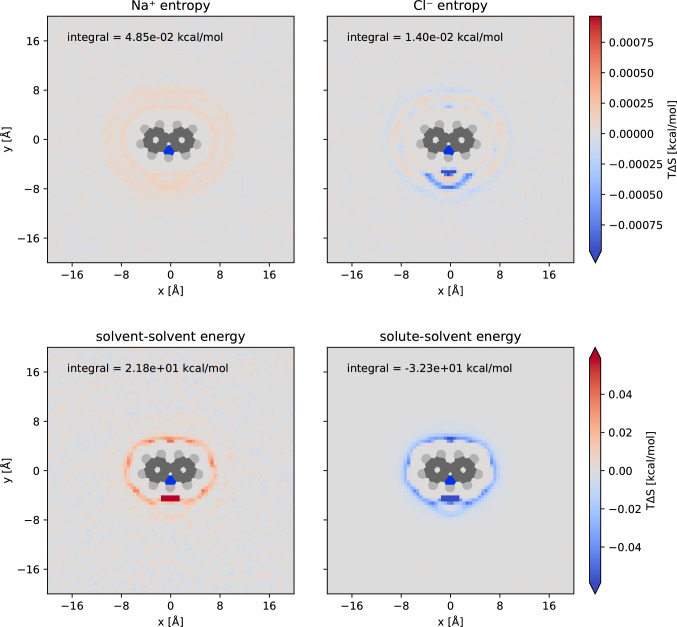
TΔS of the first order ion entropy (first row) and solvent–solvent and solute–solvent energy around carbazole (second row). To improve visibility of smaller values, the colorbar in each row is scaled so that the highest absolute value exceeds the color limit 25-fold. The integrals were computed within 10 Å of any carbazole atom

We performed a 10 μs simulation of a 1 M NaCl solution around carbazole, storing 10 million frames. We used all frames to obtain first-order ion entropies, and 100,000 frames to compute the GIST energy contributions. We use a reference density of 0.03244 ${\AA }^{-3}$ for water and $6.190\times {10}^{-4}$ and $6.145\times {10}^{-4}$ for Na^+^ and Cl^−^, respectively. We reference the energy using Eq. 11 and subtract a reference value of 0.0024 kcal/mol from $T\Delta {S}^{six}$. Figure 1 shows slices of the 3-dimensional contributions to those quantities around the carbazole.

We find that the Na^+^ entropy around carbazole is consistently positive, indicating a reduced density compared to bulk. The Cl^−^ entropy is also mostly positive but features a strongly negative region around the nitrogen atom, indicating that the density there is increased compared to bulk. In total, the first order entropy contributes very little to the solvation free energy, with integrals of 0.05 and 0.01 kcal/mol for Na^+^ and Cl^−^, respectively.

The energy contributions are much larger. As expected, we find that the solute–solvent energy is negative, while the referenced solvent–solvent energy is positive. The effect of the hydrophobic moiety on the energy contributions is much smaller than that of the nitrogen atom, which is a hydrogen bond donor. This is also true for the solvent–solvent contribution, confirming that the hydrogen bond network around a hydrophobic solvent stays largely intact, while a hydrogen bond donor reduces the number of water–water hydrogen bonds.

### Second order entropy with and without KSA

We use Eq. 14 to compute the second order (3-body) entropy of all combinations of water, Na^+^ and Cl^−^ around carbazole at 1 M salt concentration. We compute the entropy using histograms of the radial solvent–solvent distance on a three-dimensional grid. We use a grid resolution of 1 × 1 × 1 Å^3^ and a radial bin size of 0.125 Å. All entropy values shown are multiplied by ${k}_{B}T$ to give (negative) free energy contributions, and properly referenced to bulk. To assert that the bin size of 0.125 is sufficient, we re-computed the water–water, water–cation and water­–anion entropy at a coarser bin size of 0.25 Å. The result is very similar and is shown in SI Fig. 1.

**Fig. 2:**
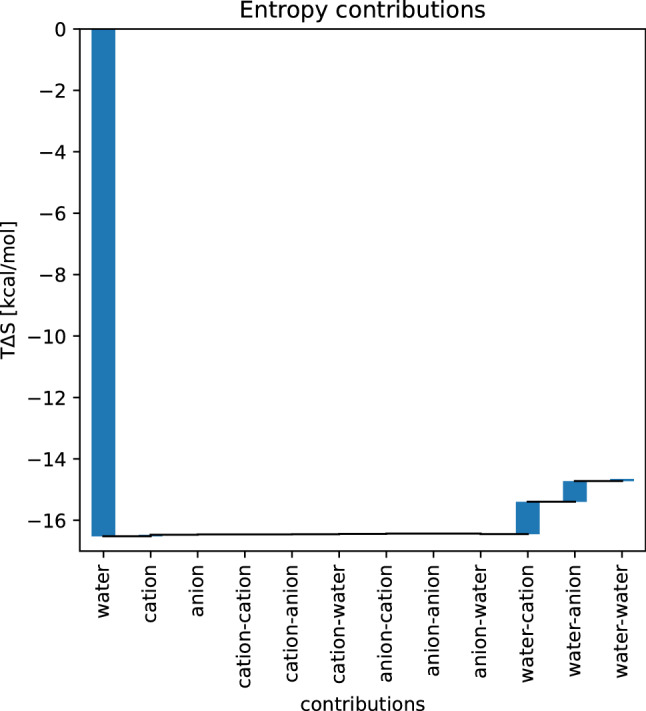
Waterfall plot showing the contributions of different entropy terms to the solvation free energy of carbazole in 1 M NaCl solution

The total contributions of all first- and second-order entropies are shown in Fig. 2. The largest contributions to the total entropy are the first order water entropy and the second order water–cation and water–anion entropies.

**Fig. 3:**
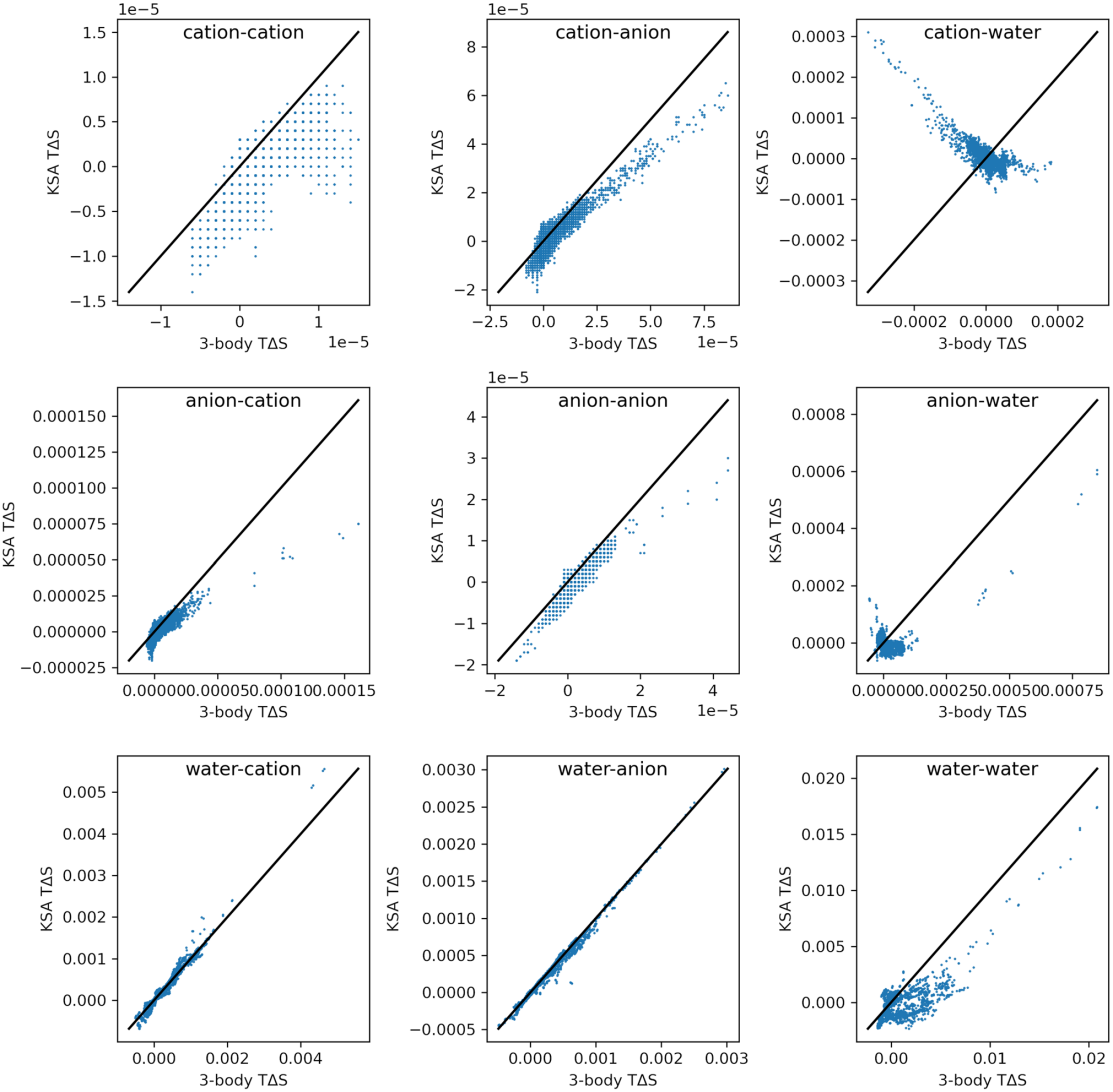
Scatter plots of the KSA entropy values per voxel against the values from the conditional histograms, for every combination of solvents. All values are in kcal/(mol Å^3^)

Furthermore, we computed all second order entropies using the KSA. This allows us to validate this approximation by plotting the KSA values per voxel against the respective values from the conditional histograms, as shown in Fig. 3.

We find that the predictions of the KSA are qualitatively wrong whenever the second molecule ${v}{^{\prime}}$ is water. This is seen most clearly in the cation–water entropy, but the correlations for the anion–water and water–water entropies are similarly bad, except for few voxels with very high entropies.

On the other hand, the entropies where ${v}{^{\prime}}$ is an ion are predicted much better by the KSA. This finding is consistent with literature stating that the KSA is valid at sufficiently low density [89]. Especially the water–cation and water–anion correlations are predicted almost perfectly. This is especially important since those contributions show very large absolute numbers compared to the ion–ion entropies.

As seen in Fig. 2, the water–water entropy shows the highest individual voxel values. However, the integral of this contribution is very small, since the positive and negative values cancel out. We note that this does not take rotational contributions into account, which are likely to be significant for the water–water entropy, due to the high anisotropy of water.

**Fig. 4:**
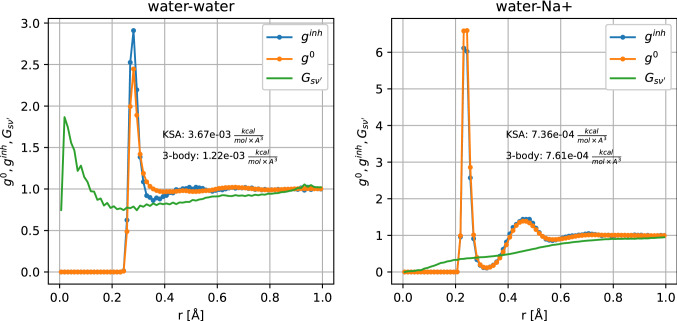
${\mathrm{g}}^{\mathrm{inh}}$, ${\mathrm{g}}^{0}$, and ${\mathrm{G}}_{\mathrm{s}{\upnu }^{{{\prime}}}}$ distribution functions for water–water (left panel) and water–Na^+^ (right panel) systems, at a grid voxel close to the carbazole molecule, centered at (0.5, 4.5, 0.5) Å in the same coordinate system as used below

To gain a better understanding of why the KSA works for the water–ion contributions but not for the water–water contribution, we plot the individual ${g}^{inh}$, ${g}^{0}$, and ${G}_{s{\nu }{^{\prime}}}$ values that are required to compute the second order entropy. Figure 4 shows the water–water and water–cation distribution functions at a grid voxel close to the carbazole molecule (centered at [0.5, 4.5, 0.5] Å). Using the KSA amounts to assuming that g^inh^ and g^0^ are equal.

In terms of GIST, the assumption underlying the KSA is that, given solvent types $\nu$ and ${\nu }{^{\prime}}$, a molecule $\nu$ in a region with little ${\nu }{^{\prime}}$ will be poorly coordinated by ${\nu }{^{\prime}}$ molecules. In the case of water–water this assumption is violated, as shown in the left panel of Fig. 4. ${G}_{s{\nu }{^{\prime}}}$ indicates that there should be rather little water at a distance of 0.3 nm to the reference voxel, since a part of the volume at this distance is excluded by the solute. Therefore, the KSA assumes that a water molecule at this position would be poorly coordinated. However, the high peak in ${g}^{inh}$ shows that this is not the case, and that a water molecule at this position is indeed coordinated rather strongly, resulting in a second-order entropy that is lower than in bulk. This is expected, since it is well known [90] that water molecules near small hydrophobic solutes tend to keep up their hydrogen bond network at the cost of an entropic penalty.

In the case of the water–cation entropy, however, the assumption of the KSA holds true. The right panel of Fig. 4 shows that the coordination of a water molecule with Na^+^ is reduced in the vicinity of a hydrophobic solute. For this specific test voxel, it is even lower than expected by the KSA, indicated by a lower peak in ${g}^{inh}$.

**Fig. 5:**
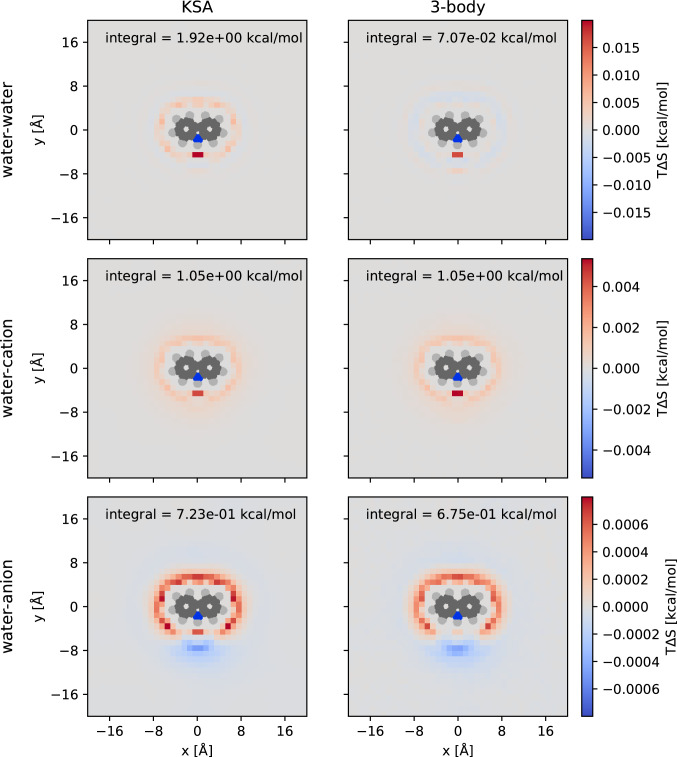
Spatially resolved water–water, water–cation, and water–anion entropies around carbazole. The left column shows the values computed from the KSA, while the right column shows the values from the conditional histogram approach. The outline of carbazole is superposed on the image for reference. The integrals were computed within 10 Å of any carbazole atom

In Fig. 5, we visualize the spatially resolved water–water, water–cation, and water–anion entropy contributions around carbazole. The carbazole molecule may be divided in two regions, where one is a large hydrophobic moiety formed by the aromatic carbon atoms, and the other is the nitrogen atom, which is a hydrogen bond donor and therefore creates a region of increased water and Cl^−^ density.

For the water–water contribution (first row in Fig. 5), we find that the KSA is qualitatively wrong over the whole hydrophobic moiety of carbazole, predicting the wrong sign of the water–water entropy contribution. Close to the hydrogen bond donor, the agreement is better. This is because the carbazole nitrogen already satisfies one of the water hydrogen bonds, and thereby reduces the coordination of water more effectively than the hydrophobic region.

In contrast, the KSA performs very well overall for the water–cation contribution (second row in Fig. 5). The highest values are found close to the hydrogen bond donor group, where the water density is very high, but the Na^+^ density is very low. Similarly, the KSA performs well for the water–anion contribution. Here, we find an additional region with entropy lower than bulk, indicating that water in this region may be coordinated to a Cl^−^ that is next to the nitrogen atom, resulting in an increased ${G}_{s{\nu }{^{\prime}}}$.

### Free energy of solvation

We performed TI calculations of carbazole in 1.0 M NaCl solution, which result in a solvation free energy of −7.4 kcal/mol. Summing up all the energies and first- and second-order entropies calculated above results in a free energy of solvation of carbazole of +4.2 kcal/mol. As shown above, the effect of the water–water entropy is very small, but it is known from previous work on GIST in pure water that the second and higher orders should be on the order of −0.4 times the first order water entropy. If we omit the second-order entropy and use this approximation instead, we arrive at a more realistic value of −2.34 kcal/mol.

This shows that orientational water–water correlations are more relevant to the final hydration free energy than the radial contribution described here. However, an analog linear correction does not seem to be possible for the ionic contributions, since e.g., the water–ion correlations are larger than the total ion entropy.

For prospective works, this implies that the water–water entropy should be estimated either via a simple linear correction, or via a higher-dimensional approximation of the second-order entropy. Since the KSA performs well on water–ion entropies, while strongly reducing the amount of sampling required, it should be feasible to compute ionic entropies via the KSA in a higher-dimensional representation. This would allow for fast and accurate estimation of the ionic entropy. A full 3-body treatment of the ionic contributions, however, would be prohibitively challenging in terms of sampling requirements. Therefore, such a higher-dimensional representation is out-of-scope for the present work.

### ***K***_S_ values from GIST

To test the validity of our method on a more diverse set of structures, we used GIST to calculate *K*_S_ values of 16 rigid small molecules, for which experimental *K*_S_ values are available from the literature [34]. We only use the rigid molecules from the dataset, since GIST is defined with respect to a rigid solute structure, and flexible molecules require individual calculations for each relevant conformation.

For each molecule, we performed 11 simulations at different salt concentrations from 0 to 1.0 M and used GIST to obtain the free energy of solvation (Δ*G*_solv_). We did not perform the full 3-body calculation but estimated the second order entropy contributions using the KSA (excluding the water–water contribution).

Δ*G*_*solv*_ increases linearly with the salt concentration. We compute *K*_S_ from the slope of Δ*G*_solv_ and use the uncertainty of the fit as a measure of accuracy.

**Fig. 6:**
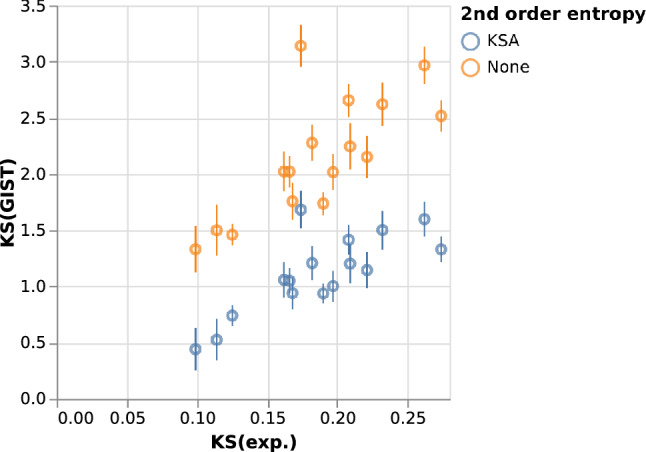
Comparison of computed KS values with experimental values. Blue: with second order entropy contributions as computed by the KSA. Orange: without second order entropy

In Fig. 6, we compare the results to experimental *K*_S_ values [34].

Without the second order entropy, we find a Pearson correlation of 0.74 between the calculated and experimental values. However, the individual values are substantially higher than the experimental ones. This shows that the missing higher order entropies counteract the energy contribution in terms of the salting out effect.

Subtracting the second order integrals of TΔS, we find that the absolute values are significantly closer to the experimental ones, and the Pearson correlation (R) slightly improves from 0.74 to 0.78. This indicates that the second order entropy, and especially the water–ion entropy, present a large negative contribution to (and thereby reduce) the salting-out effect.

There is still a significant overprediction of the salting-out effect. One likely reason for this is the simplified 1-dimensional representation of the pair distribution functions in the second-order entropy. However, higher-order entropy contributions might also play a role.

In Fig. 6, the *K*_S_ value of bisphenol A (BPA) is significantly overpredicted. This has already been noted in prior work by Misin et al. [54]. They argue that this overprediction is due to a relatively stable dimer between BPA and a Na^+^ ion, which is hard to describe using conventional force fields.

## Conclusion

The thermodynamics of solvation are crucial in many fields, such as the pharmaceutical industry or environmental research. While most implicit solvent models treat the ionic strength as an integral part of solvation, many methods based on explicit solvent models lack in this regard.

While the GIST method has been previously applied to a wide range of topics ranging from drug discovery [91, 92] to biophysical properties of antibodies [57], it was previously limited to pure solvents composed of rigid molecules. Here, we extend it towards salt-water systems and, prospectively, to arbitrary solvent mixtures. Since many biochemical processes and methods rely on high salt concentrations, we believe that this will greatly increase the applicability of GIST, and that the detailed resolution of free energy values computed by this method will be valuable for in-depth analysis of solvation effects. In cases where ions are only used to neutralize the simulation box, the ionic entropy contributions will be negligibly small due to the low concentration. However, our approach provides a physical way of treating the ion–water energy in those cases.

While the extension of the energy calculation is relatively straight-forward, the entropy calculation is more challenging. We benchmark our algorithm by presenting spatially resolved contributions to the solvation entropy of carbazole in a 1 M NaCl solution. We compute the second order entropy from a 1-dimensional histogram representation on a 3D grid and test the possibility of quickly estimating those contributions using the KSA.

Our results show that the first-order ion entropy, as well as the second order ion–ion and ion–water entropies, are very minor contributions to the solvation entropy. On the other hand, the water–ion entropies contribute strongly to the free energy of solvation. The water–water contribution largely cancels out, even though the individual voxel values are rather high. Combined with literature results on solvation in pure water showing that the higher order entropies are roughly −0.4 times the first order entropy, this indicates that orientational contributions also play a significant role.

The good performance of the KSA at computing water–ion entropies is crucial for practical applications of our method, since it allows a fast estimation of the ionic contributions to the entropy of solvation, which would otherwise be very challenging computationally. In future works, using a higher-dimensional representation of the water–ion pair distribution function for the KSA computation might also be feasible.

However, the KSA should not be used to compute the water–water entropy, and will probably also fail when the salt concentration significantly exceeds the 1 mol/L used here. Instead, the relatively high density of water makes it easier to obtain converged results for the 3-body entropy calculation. The most effective way of obtaining the full solvation entropy in a salt-water mixture would be a combination of KSA calculations for the ionic terms and full 3-body computation for the water–water entropy.

We further benchmark our algorithm by calculating salting-out coefficients for a set of 16 small molecules. We find a compelling correlation to experimental values, though the individual values are strongly overpredicted. The second order reduces this overprediction roughly by a factor of 2, indicating that the missing degrees of freedom are also relevant.

Due to the wide applicability of GIST, we expect that our extension will provide an effective tool to explain salt-dependent processes that govern the function of biomolecular systems.

## Supplementary Information

Below is the link to the electronic supplementary material.

Supplementary file1 (PDF 559 kb)

Supplementary file2 (CSV 13 kb)

## Data Availability

Initial structures for the MD simulations were obtained from PubChem. The trajectories and GIST output files are not included with this manuscript, but may be obtained from the authors.

## Code availability

The second order entropy and the first order ion entropy may be obtained from https://github.com/liedllab/second-disorder. The updated first order entropy of water is implemented in the newest version of GIGIST.

## References

[1] Ball P (2000) Nature

[2] Bachler J, Handle PH, Giovambattista N, Loerting T (2019). Phys Chem Chem Phys.

[3] Glasser L (2004). J Chem Educ.

[4] Homans WS (2007). Drug Discov Today.

[5] Coleridge ST (1798) The rime of the Ancyent Marinere. Lyrical Ballads

[6] Maurer M, Oostenbrink C (2019) J Mol Recogn 32(12):e2810 10.1002/jmr.2810 PMC6899928 31456282

[7] Qvist J, Persson E, Mattea C, Halle B (2009). Faraday Discuss.

[8] de Beer SBA, Vermeulen NPE, Oostenbrink C (2010). Curr Top Med Chem.

[9] Ball P (2008). Chem Rev.

[10] Spitzer GM, Fuchs JE, Markt P, Kirchmair J, Wellenzohn B, Langer T, Liedl KR (2008). ChemPhysChem.

[11] Xie W-H, Shiu W-Y, Mackay D (1997). Mar Environ Res.

[12] Hebditch M, Roche A, Curtis RA, Warwicker J (2019). J Pharm Sci.

[13] Sormanni P, Amery L, Ekizoglou S, Vendruscolo M, Popovic B (2017) Sci Rep 7(1):8200 10.1038/s41598-017-07800-w PMC5558012 28811609

[14] Schildkraut C, Lifson S (1965). Biopolymers.

[15] Zhang F, Liu B, Lopez A, Wang S, Liu J (2020) Nanotechnology 31(19):195503 10.1088/1361-6528/ab6fdf 31978920

[16] Zhang Y, Cremer PS (2010). Ann Rev Phys Chem.

[17] Haverick M, Mengisen S, Shameem M, Ambrogelly A (2014). MAbs.

[18] Jain T, Boland T, Lilov A, Burnina I, Brown M, Xu YD, Vasquez M (2017). Bioinformatics.

[19] Sule SV, Dickinson CD, Lu J, Chow CK, Tessier PM (2013) Mol Pharm 10(4):1322 10.1021/mp300524x 23383873

[20] Rembert KB, Paterova J, Heyda J, Hilty C, Jungwirth P, Cremer PS (2012). J Am Chem Soc.

[21] Nguyen C, Yamazaki T, Kovalenko A, Case DA, Gilson MK, Kurtzman T, Luchko T (2019) PLos One 14(7):e0219473 10.1371/journal.pone.0219473 PMC6619770 31291328

[22] Nguyen CN, Young TK, Gilson MK (2012) J Chem Phys 137(4):044101 10.1063/1.4733951 PMC3416872 22852591

[23] Setschenow J (1889). Z Phys Chem.

[24] Bockris JOM, Bowler-Reed J, Kitchener JA (1951). Trans Faraday Soc.

[25] Mcdevit WF, Long FA (1952). J Am Chem Soc.

[26] Shoor SK, Gubbins KE (1969). J Phys Chem.

[27] Chitra R, Smith PE (2001). J Phys Chem B.

[28] Kalra A, Tugcu N, Cramer SM, Garde S (2001). J Phys Chem B.

[29] van der Vegt NFA, van Gunsteren WF (2004). J Phys Chem B.

[30] Sormanni P, Aprile AF, Vendruscolo M (2015). J Mol Biol.

[31] Voynov V, Chennamsetty N, Kayser V, Helk B, Trout B (2009). MAbs.

[32] Patel BH, Paricaud P, Galindo A, Maitland GC (2003). Ind Eng Chem Res.

[33] Oleszek-Kudlak S, Grabda M, Shibata E, Eckert F, Nakamura T (2005). Environ Toxicol Chem.

[34] Endo S, Pfennigsdorff A, Goss KU (2012). Environ Sci Technol.

[35] Zhang J, Zhang H, Wu T, Wang Q, David SV (2017). J Chem Theory Comput.

[36] Swails J, York D, Roitberg A (2014). J Chem Theory Comput.

[37] Masterton WL, Lee TP (1970). J Phys Chem.

[38] Kirkwood JG, Buff FP (1951). J Chem Phys.

[39] Hummer G, Garde S, Garcia AE, Pohorille A, Pratt LR (1996). Proc Natl Acad Sci.

[40] Widom B (1982). J Phys Chem.

[41] Graziano G (2009). J Chem Eng Data.

[42] Kirkwood JG (1935). J Chem Phys.

[43] Bruckner S, Boresch S (2011). J Comput Chem.

[44] Gilson MK, Given JA, Bush BL, McCammon JA (1997). Biophys J.

[45] Li LB, Fennell CJ, Dill KA (2014). J Chem Phys.

[46] Öhlknecht C, Lier B, Petrov D, Fuchs J, Oostenbrink C (2020). J Comput Chem.

[47] Reif MM, Oostenbrink C (2014). J Comput Chem.

[48] de Ruiter A, Oostenbrink C (2020). Curr Opin Struct Biol.

[49] Ornstein LS, Zernike F (1914). Proc Koninklijke Akademie Van Wetenschappen Te Amsterdam.

[50] Lazaridis T (1998). J Phys Chem B.

[51] Kovalenko A, Hirata F (1998). Chem Phys Lett.

[52] Skyner RE, McDonagh JL, Groom CR, van Mourik T, Mitchell JBO (2015). Phys Chem Chem Phys.

[53] Joung IS, Luchko T, Case DA (2013)

[54] Misin M, Vainikka PA, Fedorov MV, Palmer DS (2016). J Chem Phys.

[55] Huggins DJ, Payne MC (2013). J Phys Chem B.

[56] Kraml J, Kamenik AS, Waibl F, Schauperl M, Liedl KR (2019). J Chem Theory Comput.

[57] Waibl F, Fernandez-Quintero ML, Kamenik AS, Kraml J, Hofer F, Kettenberger H, Georges G, Liedl KR (2021). Biophys J.

[58] Chen L, Cruz A, Roe DR, Simmonett AC, Wickstrom L, Deng N, Kurtzman T (2021) J Chem Theory Comput 17(5):2714 10.1021/acs.jctc.0c01185 PMC8119377 33830762

[59] Kraml J, Hofer F, Kamenik AS, Waibl F, Kahler U, Schauperl M, Liedl KR (2020) J Chem Inf Model 60(8):3843 10.1021/acs.jcim.0c00289 PMC7460078 32639731

[60] Young T, Abel R, Kim B, Berne BJ, Friesner RA (2007). Proc Natl Acad Sci.

[61] Haider K, Cruz A, Ramsey S, Gilson MK, Kurtzman T (2018). J Chem Theory Comput.

[62] Li Z, Lazaridis T (2012). Computing the thermodynamic contributions of interfacial water.

[63] Olson B, Cruz A, Chen LY, Ghattas M, Ji Y, Huang KH, Ayoub S, Luchko T, McKay DJ, Kurtzman T (2020). J Comput Aided Mol Des.

[64] Nguyen C, Gilson MK, Young T. Structure and Thermodynamics of Molecular Hydration via Grid Inhomogeneous Solvation Theory. 2011:arXiv:1108.4876

[65] Nguyen CN, Cruz A, Gilson MK, Kurtzman T (2014). J Chem Theory Comput.

[66] Chen L, Cruz A, Roe DR, Simmonett AC, Wickstrom L, Deng N, Kurtzman T (2021) J Chem Theory Comput 17(5):2714 10.1021/acs.jctc.0c01185 PMC8119377 33830762

[67] Nguyen CN, Kurtzman T, Gilson MK (2016). J Chem Theory Comput.

[68] Naim AB (2013). J Adv Chem.

[69] Lebowitz JL, Percus JK (1961). Phys Rev.

[70] Virtanen P, Gommers R, Oliphant TE, Haberland M, Reddy T, Cournapeau D, Burovski E, Peterson P, Weckesser W, Bright J, van der Walt SJ, Brett M, Wilson J, Millman KJ, Mayorov N, Nelson ARJ, Jones E, Kern R, Larson E, Carey CJ, Polat I, Feng Y, Moore EW, VanderPlas J, Laxalde D, Perktold J, Cimrman R, Henriksen I, Quintero EA, Harris CR, Archibald AM, Ribeiro ANH, Pedregosa F, van Mulbregt P, Contributors S (2020). Nat Methods.

[71] Bannan CC, Calabro G, Kyu DY, Mobley DL (2016). J Chem Theory Comput.

[72] Liu S, Cao S, Hoang K, Young KL, Paluch AS, Mobley DL (2016). J Chem Theory Comput.

[73] Kim S, Chen J, Cheng T, Gindulyte A, He J, He S, Li Q, Shoemaker BA, Thiessen PA, Yu B, Zaslavsky L, Zhang J, Bolton EE (2020) Nucleic Acids Res 47(D1):D1102 10.1093/nar/gky1033 PMC6324075 30371825

[74] Frisch MJ, Trucks GW, Schlegel HB, Scuseria GE, Robb MA, Cheeseman JR, Scalmani G, Barone V, Petersson GA, Nakatsuji H, Li X, Caricato M, Marenich AV, Bloino J, Janesko BG, Gomperts R, Mennucci B, Hratchian HP, Ortiz JV, Izmaylov AF, Sonnenberg JL, Williams-Young D, Ding F, Lipparini F, Egidi F, Goings J, Peng B, Petrone A, Henderson T, Ranasinghe D, Zakrzewski VG, Gao J, Rega N, Zheng G, Liang W, Hada M, Ehara M, Toyota K, Fukuda R, Hasegawa J, Ishida M, Nakajima T, Honda Y, Kitao O, Nakai H, Vreven T, Throssell K, Montgomery JAJ, Peralta JE, Ogliaro F, Bearpark MJ, Heyd JJ, Brothers EN, Kudin KN, Staroverov VN, Keith TA, Kobayashi R, Normand J, Raghavachari K, Rendell AP, Burant JC, Iyengar SS, Tomasi J, Cossi M, Millam JM, Klene M, Adamo C, Cammi R, Ochterski JW, Martin RL, Morokuma K, Farkas O, Foresman JB, Fox DJ (2016) Gaussian 16, Revision A.03: Gaussian, Inc., Wallingford CT

[75] Bayly CI, Cieplak P, Cornell W, Kollman PA (1993) J Phys Chem 97(40):10269

[76] Wang J, Wolf RM, Caldwell JW, Kollman PA, Case DA (2004). J Comput Chem.

[77] Case DA, Ben-Shalom IY, Brozell SR, Cerutti DS, Cheatham TE, Cruzeiro VWD, Darden TA, Duke RE, Ghoreishi D, Giambasu G, Giese T, Gilson MK, Gohlke H, Goetz AW, Greene D, Harris R, Homeyer N, Huang Y, Izadi S, Kovalenko A, Krasny R, Kurtzman T, Lee TS, LeGrand S, Li P, Lin C, Liu J, Luchko T, Luo R, Man V, Mermelstein DJ, Merz KM, Miao Y, Monard G, Nguyen C, Nguyen H, Onufriev A, Pan F, Qi R, Roe DR, Roitberg A, Sagui C, Schott-Verdugo S, Shen J, Simmerling CL, Smith J, Swails J, Walker RC, Wang J, Wei H, Wilson L, Wolf RM, Wu X, Xiao L, Xiong Y, York DM, Kollman PA (2019). AMBER 2019.

[78] Jorgensen WL, Chandrasekhar J, Madura JD, Impey RW, Klein ML (1983). J Chem Phys.

[79] Joung IS, Cheatham TE (2008). J Phys Chem B.

[80] Adelman SA, Doll JD (1976). J Chem Phys.

[81] Berendsen HJC, Postma JPM, Vangunsteren WF, Dinola A, Haak JR (1984). J Chem Phys.

[82] Darden T, York D, Pedersen L (1993). J Chem Phys.

[83] Ryckaert J-P, Ciccotti G, Berendsen HJC (1977). J Comput Phys.

[84] Nguyen C, Gilson MK, Young T (2011) Structure and thermodynamics of molecular hydration via grid inhomogeneous solvation theory. arXiv:1108.4876 10.1063/1.4733951 PMC3416872 22852591

[85] Ramsey S, Nguyen C, Salomon-Ferrer R, Walker RC, Gilson MK, Kurtzman T (2016). J Comput Chem.

[86] Beutler TC, Mark AE, Van Schaik RC, Gerber PR, Van Gunsteren WF (1994). Chem Phys Lett.

[87] Steinbrecher T, Joung I, Case DA (2011). J Comput Chem.

[88] Chodera JD, Swope WC, Pitera JW, Seok C, Dill KA (2007) J Chem Theory Comput 3(1):26 10.1021/ct0502864 26627148

[89] Singer A (2004). J Chem Phys.

[90] Hande VR, Chakrabarty S (2015). J Phys Chem B.

[91] Balius TE, Fischer M, Stein RM, Adler TB, Nguyen CN, Cruz A, Gilson MK, Kurtzman T, Shoichet BK (2017). Proc Natl Acad Sci USA.

[92] Hufner-Wulsdorf T, Klebe G (2021). J Med Chem.

