# Supplementary material for: Explicit solvation thermodynamics in ionic solution: extending grid inhomogeneous solvation theory to solvation free energy of salt–water mixtures
Source: J Comput Aided Mol Des. 2022 Jan 15;36(2):101–16. doi: 10.1007/s10822-021-00429-y (PMC8907097; doi:10.1007/s10822-021-00429-y)
Supplement: Supplementary file 1 — Supplementary file1 (PDF 559 kb) [file 10822_2021_429_MOESM1_ESM.pdf]

Supporting Information

Explicit Solvation Thermodynamics in Ionic Solution

Extending Grid Inhomogeneous Solvation Theory Computes To Solvation Free Energy of Salt-Water Mixtures

Convergence of 2nd order entropies with respect to the bin spacing

To test whether the bin spacing of 0.125 Å in g^{inh} is sufficient, we re-binned the values to 0.25 Å and computed the second order entropy. The result is shown in SI Figure 1.

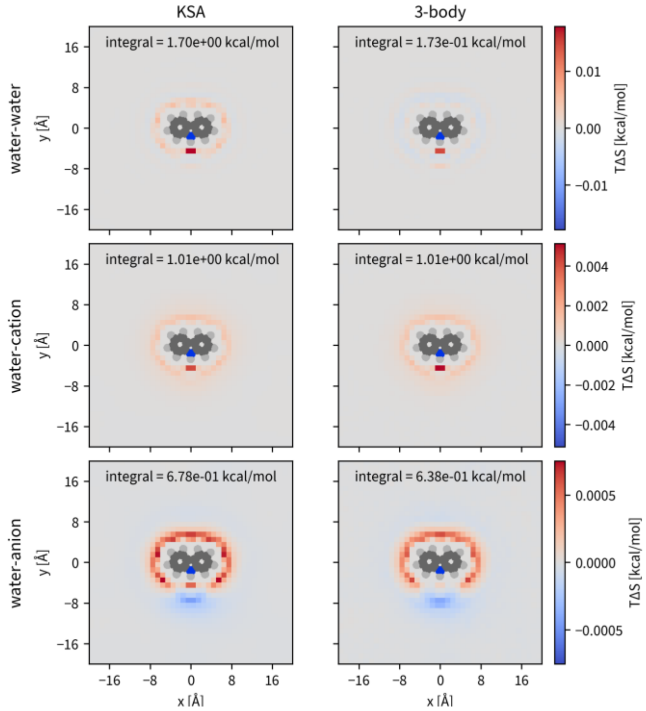

Figure 1: Spatially resolved water-water, water-cation, and water-anion entropies around carbazole, as in Figure 5 of the main text, but computed at a bin spacing of 0.25 Å.

We find that the results are qualitatively similar in all cases. Furthermore, the integrals are very close for the water-ion entropies. The biggest differences are in the water-water entropy, with a difference of 0.22 and 0.1 kcal/mol to the results from the finer bin spacing. Since the positive and negative values cancel out for the water-water 3-body calculation, the error is bigger than the absolute value of the integral in this case (with an integral of 0.07 kcal/mol).
However, since the values are always qualitatively similar, and the quantitative agreement is very good except for the water-water 3-body calculation, we conclude that the (finer) bin spacing of 0.125 Å should be sufficient for this study.

Salting-Out coefficients from the free energy of solvation

The Salting-Out coefficient K_S describes the dependence of the solubility of a compound on the salt concentration. It is defined via the Setschenow equation:

$$\log \frac{S}{S_0} = -K_S \times c_{\text{salt}} \tag{1}$$

Here, S and S_0 describe the solubility of the compound at the salt concentration c_{salt} and in pure water, respectively.

We further express the solubility in terms of the free energy of solvation:

$$S = \exp\left(-\frac{\Delta G_{\text{solv}}}{RT}\right) \tag{2}$$

Here, ΔG_{solv} denotes the change in free energy upon placing a solute molecule from the gas phase into the solution, R denotes the gas constant, and T the temperature. Note that, while ΔG_{solv} is defined using the gas phase as a reference, our approach is equally valid for solid substances, since we will only be working with differences between ΔG_{solv} at varying conditions in the solute phase, such that the properties of the reference state cancel out.

Further note that we neglect any solute-solute interactions in the liquid phase. This will generally be valid at low solubilities but might lead to errors with highly soluble compounds.

Combining Equations 12 and 13, we rewrite the Setschenow equation as:

$$\log \frac{S}{S_0} = \frac{\Delta G^0_{\text{solv}} - \Delta G_{\text{solv}}(c_{\text{salt}})}{RT \ln 10} = -K_S \times c_{\text{salt}} \tag{3}$$

Which leads us to the linearized equation:

$$\Delta G_{\text{solv}}(c_{\text{salt}}) = \Delta G^0_{\text{solv}} + RT \ln 10\, K_S \times c_{\text{salt}} \tag{4}$$

Re-computed salt concentration

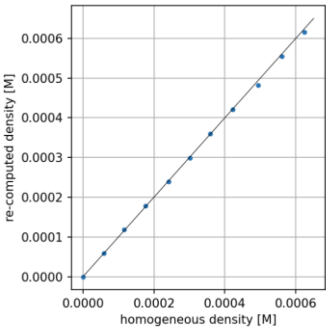

Figure 2: Plot of the recomputed ion density against the density of a homogeneous system with the solute removed, but equal composition of the solvent. Based on the simulations of caffeine in NaCl at salt concentrations from 0.0 to 1.0M.

To calculate the salting-out coefficient K_S, the salt concentration is needed. The average salt concentration of the system corresponds to g^0 in the IST formalism. However, the density distribution G_ν is normalized with respect to the bulk concentration g^∞. Therefore, we compute salt concentrations from g^∞ and compare to the average salt concentration of the system. SI Figure 2 shows that the difference is very minor.

Inhomogeneous Solvation Theory (IST) with multiple solvents

Here, we present a formal extension of IST to multiple solvents. We attempt to stay as close to the original derivation by Lazaridis [1] as possible, and focus on the differences due to the extension to mixtures.

Lazaridis starts by citing equations by Morita and Hiroike for the free energy of liquids given an external potential. [2] The same work also contains generalized equations for mixtures. Here, $\boldsymbol{x}$ is used to describe the coordinates of multiple species, i.e., $d\boldsymbol{x} = \sum_\nu d\boldsymbol{r}$. v is defined as $1 - g(x, x')$,

where g is the canonical pair distribution function. λ is the thermal de Broglie wavelength ($\lambda = (h^2/2\pi mkT)^{\frac{1}{2}}$, see [3])and affects the ideal gas contribution to the absolute free energy. Furthermore, ρ is the density, u is the external potential (which will be used to describe the solute), k is Boltzmann's constant, and T is the temperature.

$$\begin{aligned}\frac{A}{kT} = \int d\boldsymbol{x}\frac{\rho(\boldsymbol{x})u_{sv}(\boldsymbol{x})}{kT} - \frac{1}{2}\int\int d\boldsymbol{x}\, d\boldsymbol{x}'\rho(\boldsymbol{x})\rho(\boldsymbol{x}')\big(1+v(\boldsymbol{x},\boldsymbol{x}')\big)\ln\big(1+b(\boldsymbol{x},\boldsymbol{x}')\big) \\ + \int d\boldsymbol{x}\,\rho(\boldsymbol{x})(\ln \lambda^3\,\rho(\boldsymbol{x})-1) \\ + \frac{1}{2}\int\int d\boldsymbol{x}\, d\boldsymbol{x}'\rho(\boldsymbol{x})\rho(\boldsymbol{x}')\big[\big(1+v(\boldsymbol{x},\boldsymbol{x}')\big)\ln\big(1+v(\boldsymbol{x},\boldsymbol{x}')\big)-v(\boldsymbol{x},\boldsymbol{x}')\big]\end{aligned} \tag{5}$$

$-$ 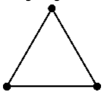 $+$ 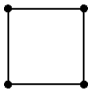 $- \cdots -$ all more than doubly connected diagrams

We proceed by substituting $1 + v(x, x') = g(x, x')$ and $1 + b(\boldsymbol{x}, \boldsymbol{x}') = \exp(-u(\boldsymbol{x}, \boldsymbol{x}')/\mathrm{kT})$. Furthermore, we use $\int \rho(x)dx = \bar{N}$. This leads us to:

$$\begin{aligned}\frac{A}{kT} \\ = \int d\boldsymbol{x}\frac{\rho(\boldsymbol{x})u_{sv}(\boldsymbol{x})}{kT} + \frac{1}{2}\int\int d\boldsymbol{x}\, d\boldsymbol{x}'\frac{\rho(\boldsymbol{x})\rho(\boldsymbol{x}')g(\boldsymbol{x},\boldsymbol{x}')u(x,x')}{kT} - \bar{N} + \int d\boldsymbol{x}\,\rho(\boldsymbol{x})\ln(\lambda^3\rho(\boldsymbol{x})) \\ + \frac{1}{2}\int\int d\boldsymbol{x}\, d\boldsymbol{x}'\rho(\boldsymbol{x})\rho(\boldsymbol{x}')\big[g(\boldsymbol{x},\boldsymbol{x}')\ln\big(g(\boldsymbol{x},\boldsymbol{x}')\big)-g(\boldsymbol{x},\boldsymbol{x}')+1\big]\end{aligned} \tag{6}$$

$-$ 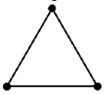 $+$ 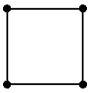 $- \cdots$

$-$ all more than doubly connected diagrams of black circles (ρ) and (g−1) bonds.

In the next step, we split the free energy into energy and entropy contributions. Note that there is an additional $\frac{3}{2}\bar{N}kT$ term describing the kinetic energy. We will omit the higher-order terms (represented by the diagrams) but note that they would be part of the entropy term.

$$E = \frac{3}{2}\bar{N}kT + \int d\boldsymbol{x}\rho(\boldsymbol{x})u_{sv}(\boldsymbol{x}) + \frac{1}{2}\int\int d\boldsymbol{x}\, d\boldsymbol{x}'\rho(\boldsymbol{x})\rho(\boldsymbol{x}')g(\boldsymbol{x},\boldsymbol{x}')u(x,x') \tag{7}$$

$$\begin{aligned}\frac{S}{k} = \frac{5}{2}\bar{N} - \int d\boldsymbol{x}\,\rho(\boldsymbol{x})\ln(\lambda^3\rho(\boldsymbol{x})) \\ - \frac{1}{2}\int\int d\boldsymbol{x}\, d\boldsymbol{x}'\rho(\boldsymbol{x})\rho(\boldsymbol{x}')\big[g(\boldsymbol{x},\boldsymbol{x}')\ln\big(g(\boldsymbol{x},\boldsymbol{x}')\big)-g(\boldsymbol{x},\boldsymbol{x}')+1\big]\end{aligned} \tag{8}$$

In the following, we will explicitly write sums over the contributions of each species v. The following substitutions apply:

$$\boldsymbol{x} \rightarrow \text{coordinates } \boldsymbol{r}\text{, species } \nu \tag{9}$$

$$\int d\boldsymbol{x} = \sum_{\nu}\int d\boldsymbol{r} \tag{10}$$

This leads us to:

$$E = \frac{3}{2}\bar{N}kT + \sum_{\nu}\int d\boldsymbol{r}\,\rho_\nu(\boldsymbol{r})u_{sv}(\boldsymbol{r}) + \frac{1}{2}\sum_{\nu}\sum_{\nu'}\int\int d\boldsymbol{r}\, d\boldsymbol{r}'\rho_\nu(\boldsymbol{r})\rho_{\nu'}(\boldsymbol{r}')g_{\nu,\nu'}(\boldsymbol{r},\boldsymbol{r}')u(\boldsymbol{r},\boldsymbol{r}') \tag{11}$$

$$\frac{S}{k} = \frac{5}{2}\bar{N} - \sum_{\nu} \int d\boldsymbol{r}\, \rho_\nu(\boldsymbol{r}) ln({\lambda_\nu}^3 \rho_\nu(\boldsymbol{x})) - \frac{1}{2}\sum_{\nu}\sum_{\nu'} \int\int d\boldsymbol{r}\, d\boldsymbol{r}' \rho_\nu(\boldsymbol{r})\rho_{\nu'}(\boldsymbol{r}') \left[g_{\nu,\nu'}(\boldsymbol{r},\boldsymbol{r}')\, ln\left(g_{\nu,\nu'}(\boldsymbol{r},\boldsymbol{r}')\right) - g_{\nu,\nu'}(\boldsymbol{r},\boldsymbol{r}') + 1\right] \quad (12)$$

In the grand canonical ensemble (GCE), the PCF (pair correlation function) tends towards 1 in the limit of long separation between the two atoms of interest. In the canonical ensemble (CE), however, the PCF does not converge to 1. The reason is that, by fixing the position of a single particle in space, the volume left to the other particles is modified. Therefore, Lazaridis defines a new PCF in terms of the density at long distance from the solute. This is roughly equivalent to the PCF in the GCE.

$$\rho_\nu(r) \rightarrow \rho_\nu^\infty G_{s\nu}(r) \quad (13)$$

$$g_{\nu,\nu'}(r,r') \rightarrow g_{\nu,\nu'}^{inh}(r,r') \quad (14)$$

Substituting in the new PCF and the limiting density ρ^∞ in above equations, we obtain

$$E = \frac{3}{2}\bar{N}kT + \sum_{\nu} \rho_\nu^\infty \int d\boldsymbol{r}\, G_{s\nu}(\boldsymbol{r}) u_{s\nu}(\boldsymbol{r}) + \frac{1}{2}\sum_{\nu}\sum_{\nu'} \rho_\nu^\infty \rho_{\nu'}^\infty \int\int d\boldsymbol{r}\, d\boldsymbol{r}' G_{s\nu}(\boldsymbol{r}) G_{s\nu'}(\boldsymbol{r}') g_{\nu,\nu'}^{inh}(\boldsymbol{r},\boldsymbol{r}') u_{\nu\nu'}(\boldsymbol{r},\boldsymbol{r}') \quad (15)$$

$$\frac{S}{k} = \frac{5}{2}\bar{N} - \sum_{\nu} \int d\boldsymbol{r}\, \rho_\nu^\infty G_{s\nu}(\boldsymbol{r}) ln({\lambda_\nu}^3 \rho_\nu^\infty G_{s\nu}(\boldsymbol{r})) - \frac{1}{2}\sum_{\nu}\sum_{\nu'} \rho_\nu^\infty \rho_{\nu'}^\infty \int\int d\boldsymbol{r}\, d\boldsymbol{r}' G_{s\nu}(\boldsymbol{r}) G_{s\nu'}(\boldsymbol{r}') \left[g_{\nu,\nu'}^{inh}(\boldsymbol{r},\boldsymbol{r}')\, ln\left(g_{\nu,\nu'}^{inh}(\boldsymbol{r},\boldsymbol{r}')\right) - g_{\nu,\nu'}^{inh}(\boldsymbol{r},\boldsymbol{r}') + 1\right] \quad (16)$$

We further split the constant and r-dependent terms in the logarithm in the 2nd term of S:

$$\frac{S}{k} = \frac{5}{2}\bar{N} - \sum_{\nu} \int d\boldsymbol{r}\, \rho_\nu^\infty G_{s\nu}(\boldsymbol{r}) ln({\lambda_\nu}^3 \rho_\nu^\infty) - \sum_{\nu} \int d\boldsymbol{r} \rho_\nu^\infty G_{s\nu}(\boldsymbol{r})\, ln\left(G_{s\nu}(\boldsymbol{r})\right) - \frac{1}{2}\sum_{\nu}\sum_{\nu'} \rho_\nu^\infty \rho_{\nu'}^\infty \int\int d\boldsymbol{r}\, d\boldsymbol{r}' G_{s\nu}(\boldsymbol{r}) G_{s\nu'}(\boldsymbol{r}') \left[g_{\nu,\nu'}^{inh}(\boldsymbol{r},\boldsymbol{r}')\, ln\left(g_{\nu,\nu'}^{inh}(\boldsymbol{r},\boldsymbol{r}')\right) - g_{\nu,\nu'}^{inh}(\boldsymbol{r},\boldsymbol{r}') + 1\right] \quad (17)$$

Using $\int d\boldsymbol{r}\, \rho_v^\infty G_{sv}(r) = N_v$, we can simplify the second term of the solvent entropy:

$$\frac{S}{k} = \frac{5}{2}\bar{N} - \sum_v N_v ln(\lambda_v{}^3 \rho_v^\infty) - \sum_v \int d\boldsymbol{r} \rho_v^\infty G_{sv}(\boldsymbol{r})\, ln\big(G_{sv}(\boldsymbol{r})\big) - \frac{1}{2}\sum_v \sum_{v'} \rho_v^\infty \rho_{v'}^\infty \int \int d\boldsymbol{r}\, d\boldsymbol{r}' G_{sv}(\boldsymbol{r}) G_{sv'}(\boldsymbol{r}') \Big[g_{v,v'}^{inh}(\boldsymbol{r},\boldsymbol{r}')\, ln\left(g_{v,v'}^{inh}(\boldsymbol{r},\boldsymbol{r}')\right) - g_{v,v'}^{inh}(\boldsymbol{r},\boldsymbol{r}') + 1\Big] \tag{18}$$

In the next step, we subtract the energy and entropy of the unperturbed solvent. These equations describe the energy and entropy that the atoms would have if they were transferred to bulk. Note that the bulk density g^0 occurs in those equations, which is not necessarily equal to the limiting density g^∞.

$$E_{bulk} = \frac{3}{2}\bar{N}kT + \frac{1}{2}\sum_v N_v \sum_{v'} \rho_{v'}^0 \int g_{vv'}^0(\boldsymbol{r}') u_{vv'}(\boldsymbol{r}') d\boldsymbol{r}' \tag{19}$$

$$\frac{S_{bulk}}{k} = \frac{5}{2}\bar{N} - \sum_v N_v ln(\lambda_v{}^3 \rho_v^0) - \frac{1}{2}\sum_v \sum_{v'} N_v \rho_{v'}^0 \int g_{vv'}^0(\boldsymbol{r}')\, ln\, g_{vv'}^0(\boldsymbol{r}') - g_{vv'}^0(\boldsymbol{r}') + 1\, d\boldsymbol{r}' \tag{20}$$

Subtracting the bulk values leads us to the partial molar energy and entropy.

$$\bar{e}^\infty = \sum_v \rho_v^\infty \int d\boldsymbol{r}\, G_{sv}(\boldsymbol{r}) u_{sv}(\boldsymbol{r}) + \frac{1}{2}\sum_v \sum_{v'} \rho_v^\infty \rho_{v'}^\infty \int\int d\boldsymbol{r}\, d\boldsymbol{r}' G_{sv}(\boldsymbol{r}) G_{sv'}(\boldsymbol{r}') g_{v,v'}^{inh}(\boldsymbol{r},\boldsymbol{r}') u_{vv'}(\boldsymbol{r},\boldsymbol{r}') - \frac{1}{2}\sum_v N_v \sum_{v'} \rho_{v'}^0 \int g_{vv'}^0(\boldsymbol{r}') u_{vv'}(\boldsymbol{r}') d\boldsymbol{r}' \tag{21}$$

$$\frac{\bar{s}^\infty}{k} = \sum_v N_v ln(\lambda_v{}^3 \rho_v^0) - \sum_v N_v ln(\lambda_v{}^3 \rho_v^\infty) - \sum_v \int d\boldsymbol{r} \rho_v^\infty G_{sv}(\boldsymbol{r})\, ln\big(G_{sv}(\boldsymbol{r})\big) - \frac{1}{2}\sum_v \sum_{v'} \rho_v^\infty \rho_{v'}^\infty \int\int d\boldsymbol{r}\, d\boldsymbol{r}' G_{sv}(\boldsymbol{r}) G_{sv'}(\boldsymbol{r}') \Big[g_{v,v'}^{inh}(\boldsymbol{r},\boldsymbol{r}')\, ln\left(g_{v,v'}^{inh}(\boldsymbol{r},\boldsymbol{r}')\right) - g_{v,v'}^{inh}(\boldsymbol{r},\boldsymbol{r}') + 1\Big] + \frac{1}{2}\sum_v \sum_{v'} N_v \rho_{v'}^0 \int g_{vv'}^0(\boldsymbol{r}')\, ln\, g_{vv'}^0(\boldsymbol{r}') - g_{vv'}^0(\boldsymbol{r}') + 1\, d\boldsymbol{r}' \tag{22}$$

The main difference between IST theory of mixtures and the original formulation is the treatment of the density correction, which stems from the first 2 terms in the partial molar entropy. Lazaridis assumes that the difference between ρ^∞ and ρ^0 is due to the "thermal volume" κkT, where κ is the compressibility of the solvent. In the case of mixtures, however, there are additional differences due to different interactions with the solute, which change the composition of the solute around the solvent. These differences are typically much larger than the thermal volume assumed by Lazaridis. Furthermore, they must be evaluated for each solvent species separately. We note that this is not a

theoretical limitation of our method, because one can simply increase the total system volume until the differences become irrelevant. However, more care is required than with a single solvent.

We express the density difference in terms of the excluded volume $V_{ex,v}$ of each species v. The value of $V_{ex,v}$ depends on the exact choice of reference system. If the reference system is defined by removing the central solute particle from the solution at constant pressure and constant number of solvent particles, $V_{ex,v}$ is

$$V_{ex,v} = -\Delta V + \int 1 - G_{sv}(\boldsymbol{r})d\boldsymbol{r} \tag{23}$$

where ΔV is the change in system volume upon removal of the solute. If we set $V_{ex,v}$ to $-\kappa kT$, our derivation would lead to the same results as Lazaridis'.

Using $\rho^{\infty} \approx \rho^{0}\left(1 + \frac{V_{ex,v}}{V}\right)$ and $\ln(x) \approx x - 1$, the first two entropy terms simplify to:

$$\sum_{v} N_v ln({\lambda_v}^3 \rho_v^0) - \sum_{v} N_v ln({\lambda_v}^3 \rho_v^{\infty}) = \sum_{v} N_v \, ln\left(\frac{\rho_v^0}{\rho_v^{\infty}}\right) \approx -\sum_{v} N_v \left(\frac{V_{ex,v}}{V}\right) = -\sum_{v} \rho_v V_{ex,v} \tag{24}$$

$$\begin{aligned}\frac{\bar{s}^{\infty}}{k} = &-\sum_{v} \rho_v V_{ex,v} + \sum_{v} \int d\boldsymbol{r} \rho_v^{\infty} G_{sv}(\boldsymbol{r}) \, ln\big(G_{sv}(\boldsymbol{r})\big) \\ &-\frac{1}{2}\sum_{v}\sum_{v'} \rho_v^{\infty}\rho_{v'}^{\infty} \int\int d\boldsymbol{r}\, d\boldsymbol{r}' G_{sv}(\boldsymbol{r}) G_{sv'}(\boldsymbol{r}') \left[g_{v,v'}^{inh}(\boldsymbol{r},\boldsymbol{r}') \, ln\left(g_{v,v'}^{inh}(\boldsymbol{r},\boldsymbol{r}')\right)\right. \\ &\left. - g_{v,v'}^{inh}(\boldsymbol{r},\boldsymbol{r}') + 1\right] + \frac{1}{2}\sum_{v}\sum_{v'} N_v \rho_{v'}^0 \int g_{vv'}^0(\boldsymbol{r}') \, ln\, g_{vv'}^0(\boldsymbol{r}') - g_{vv'}^0(\boldsymbol{r}') + 1 \, d\boldsymbol{r}'\end{aligned} \tag{25}$$

We then add the kinetic energy and one-particle entropy of the solute:

$$e_s = \frac{3}{2}kT \tag{26}$$

$$\frac{s_s}{k} = \frac{3}{2} - \frac{ln(\rho_s \lambda_s^3)}{k} \tag{27}$$

This leads us to:

$$\begin{aligned}\bar{e}_s^{\infty} = &\frac{3}{2}kT + \sum_{v} \rho_v^{\infty} \int d\boldsymbol{r}\, G_{sv}(\boldsymbol{r}) u_{sv}(\boldsymbol{r}) \\ &+\frac{1}{2}\sum_{v}\sum_{v'} \rho_v^{\infty}\rho_{v'}^{\infty} \int\int d\boldsymbol{r}\, d\boldsymbol{r}' G_{sv}(\boldsymbol{r}) G_{sv'}(\boldsymbol{r}') g_{v,v'}^{inh}(\boldsymbol{r},\boldsymbol{r}') u_{vv'}(\boldsymbol{r},\boldsymbol{r}') \\ &-\frac{1}{2}\sum_{v} N_v \sum_{v'} \rho_{v'}^0 \int g_{vv'}^0(\boldsymbol{r}') u_{vv'}(\boldsymbol{r}') d\boldsymbol{r}'\end{aligned} \tag{28}$$

$$\frac{\bar{s}^{\infty}}{k} = \frac{3}{2} - \frac{ln(\rho_s \lambda_s^3)}{k} - \sum_{\nu} \rho_\nu V_{ex,\nu} - \sum_{\nu} \int d\boldsymbol{r} \rho_\nu^\infty G_{s\nu}(\boldsymbol{r}) \, ln\big(G_{s\nu}(\boldsymbol{r})\big)$$
$$- \frac{1}{2}\sum_{\nu}\sum_{\nu'} \rho_\nu^\infty \rho_{\nu'}^\infty \int \int d\boldsymbol{r}\, d\boldsymbol{r}' G_{s\nu}(\boldsymbol{r}) G_{s\nu'}(\boldsymbol{r}') \Big[g_{\nu,\nu'}^{inh}(\boldsymbol{r},\boldsymbol{r}') \, ln\Big(g_{\nu,\nu'}^{inh}(\boldsymbol{r},\boldsymbol{r}')\Big) \qquad (29)$$
$$- g_{\nu,\nu'}^{inh}(\boldsymbol{r},\boldsymbol{r}') + 1\Big] + \frac{1}{2}\sum_{\nu}\sum_{\nu'} N_\nu \rho_{\nu'}^0 \int g_{\nu\nu'}^0(\boldsymbol{r}) \, ln \, g_{\nu\nu'}^0(\boldsymbol{r}') - g_{\nu\nu'}^0(\boldsymbol{r}') + 1 \, d\boldsymbol{r}'$$

We use the identity $\int G_{s\nu} dr = \frac{V \rho_\nu}{\rho_\nu^\infty}$ to replace $N_\nu = V \rho_\nu = \rho_\nu^\infty \int G_{s\nu} dr$. Furthermore, we substitute $\rho_\nu^0 \approx \rho_\nu^\infty - \frac{{\rho_\nu^0}^2 V_{ex,\nu}}{N_\nu}$, as well as $\rho_\nu^0 \approx \frac{N_\nu}{N_{\nu'}} \rho_{\nu'}^0$. While the latter is an additional approximation compared to Lazaridis derivation, it is on the same order of magnitude as other approximations taken, since it is proportional to the difference between ρ^∞ and ρ^0, and will be multiplied only by a constant term independent of the system size (the volume $V_{ex,\nu}$ depends on the properties of the solute and solvents, but not on the system size).
For the energy:

$$-\frac{1}{2}\sum_{\nu} N_\nu \sum_{\nu'} \rho_{\nu'}^0 \int g_{\nu\nu'}^0(\boldsymbol{r}') u_{\nu\nu'}(\boldsymbol{r}') d\boldsymbol{r}'$$
$$= -\frac{1}{2}\sum_{\nu} \rho_\nu^\infty \int G_{s\nu} dr \sum_{\nu'} \left(\rho_{\nu'}^\infty - \frac{{\rho_{\nu'}^0}^2 V_{ex,\nu}}{N_{\nu'}} \right) \int g_{\nu\nu'}^0(\boldsymbol{r}') u_{\nu\nu'}(\boldsymbol{r}') d\boldsymbol{r}'$$
$$= -\frac{1}{2}\sum_{\nu} \rho_\nu^\infty \int G_{s\nu} dr \sum_{\nu'} \rho_{\nu'}^\infty \int g_{\nu\nu'}^0(\boldsymbol{r}') u_{\nu\nu'}(\boldsymbol{r}') d\boldsymbol{r}' \qquad (30)$$
$$+ \frac{1}{2}\sum_{\nu} N_\nu \sum_{\nu'} \frac{{\rho_{\nu'}^0}^2 V_{ex,\nu}}{N_{\nu'}} \int g_{\nu\nu'}^0(\boldsymbol{r}') u_{\nu\nu'}(\boldsymbol{r}') d\boldsymbol{r}'$$
$$= -\frac{1}{2}\sum_{\nu} \rho_\nu^\infty \int G_{s\nu} d\boldsymbol{r} \sum_{\nu'} \rho_{\nu'}^\infty \int g_{\nu\nu'}^0(\boldsymbol{r}') u_{\nu\nu'}(\boldsymbol{r}') d\boldsymbol{r}'$$
$$+ \frac{1}{2}\sum_{\nu}\sum_{\nu'} \rho_\nu^0 \rho_{\nu'}^0 V_{ex,\nu} \int g_{\nu\nu'}^0(\boldsymbol{r}') u_{\nu\nu'}(\boldsymbol{r}') d\boldsymbol{r}'$$

For the entropy:

$$
\begin{aligned}
\frac{1}{2}\sum_{\nu}\sum_{\nu'} N_\nu \rho_{\nu'}^0 \int g_{\nu\nu'}^0(\boldsymbol{r'}) \ln g_{\nu\nu'}^0(\boldsymbol{r'}) - g_{\nu\nu'}^0(\boldsymbol{r'}) + 1\, d\boldsymbol{r'} \\
&= \frac{1}{2}\sum_{\nu}\sum_{\nu'} \rho_\nu^\infty \int G_{s\nu} d\boldsymbol{r}\ \rho_{\nu'}^0 \int g_{\nu\nu'}^0(\boldsymbol{r'}) \ln g_{\nu\nu'}^0(\boldsymbol{r'}) - g_{\nu\nu'}^0(\boldsymbol{r'}) + 1\, d\boldsymbol{r'} \\
&= \frac{1}{2}\sum_{\nu}\sum_{\nu'} \rho_\nu^\infty \int G_{s\nu} d\boldsymbol{r} \left(\rho_{\nu'}^\infty - \frac{{\rho_{\nu'}^0}^2 V_{ex,\nu}}{N_{\nu'}}\right) \int g_{\nu\nu'}^0(\boldsymbol{r'}) \ln g_{\nu\nu'}^0(\boldsymbol{r'}) - g_{\nu\nu'}^0(\boldsymbol{r'}) \\
&+ 1\, d\boldsymbol{r'} \\
&= \frac{1}{2}\sum_{\nu}\sum_{\nu'} \rho_\nu^\infty \int G_{s\nu} d\boldsymbol{r}\ \rho_{\nu'}^\infty \int g_{\nu\nu'}^0(\boldsymbol{r'}) \ln g_{\nu\nu'}^0(\boldsymbol{r'}) - g_{\nu\nu'}^0(\boldsymbol{r'}) + 1\, d\boldsymbol{r'} \\
&- \frac{1}{2}\sum_{\nu}\sum_{\nu'} N_\nu \frac{{\rho_{\nu'}^0}^2 V_{ex,\nu}}{N_{\nu'}} \int g_{\nu\nu'}^0(\boldsymbol{r'}) \ln g_{\nu\nu'}^0(\boldsymbol{r'}) - g_{\nu\nu'}^0(\boldsymbol{r'}) + 1\, d\boldsymbol{r'} \\
&= \frac{1}{2}\sum_{\nu}\sum_{\nu'} \rho_\nu^\infty \int G_{s\nu} d\boldsymbol{r}\ \rho_{\nu'}^\infty \int g_{\nu\nu'}^0(\boldsymbol{r'}) \ln g_{\nu\nu'}^0(\boldsymbol{r'}) - g_{\nu\nu'}^0(\boldsymbol{r'}) + 1\, d\boldsymbol{r'} \\
&- \frac{1}{2}\sum_{\nu}\sum_{\nu'} \rho_\nu^0 \rho_{\nu'}^0 V_{ex,\nu} \int g_{\nu\nu'}^0(\boldsymbol{r'}) \ln g_{\nu\nu'}^0(\boldsymbol{r'}) - g_{\nu\nu'}^0(\boldsymbol{r'}) + 1\, d\boldsymbol{r'}
\end{aligned} \tag{31}
$$

Inserting equations 30 and 31 in equation 28 and 29, the energy expression becomes:

$$
\begin{aligned}
\bar{e}_s^\infty = \frac{3}{2}kT + \sum_{\nu} \rho_\nu^\infty \int d\boldsymbol{r}\, G_{s\nu}(\boldsymbol{r}) u_{s\nu}(\boldsymbol{r}) \\
+ \frac{1}{2}\sum_{\nu}\sum_{\nu'} \rho_\nu^\infty \rho_{\nu'}^\infty \int\int d\boldsymbol{r}\, d\boldsymbol{r'} G_{s\nu}(\boldsymbol{r}) \big[G_{s\nu'}(\boldsymbol{r'}) g_{\nu,\nu'}^{inh}(\boldsymbol{r},\boldsymbol{r'}) \\
- g_{\nu\nu'}^0(\boldsymbol{r},\boldsymbol{r'})\big] u_{\nu\nu'}(\boldsymbol{r},\boldsymbol{r'}) + \frac{1}{2}\sum_{\nu}\sum_{\nu'} \rho_\nu^0 \rho_{\nu'}^0 V_{ex,\nu} \int g_{\nu\nu'}^0(\boldsymbol{r'}) u_{\nu\nu'}(\boldsymbol{r'}) d\boldsymbol{r'}
\end{aligned} \tag{32}
$$

And the entropy becomes

$$
\begin{aligned}
\frac{\bar{s}^\infty}{k} = \frac{3}{2} - \frac{\ln(\rho_s \lambda_s^3)}{k} - \sum_{\nu} \rho_\nu V_{ex,\nu} - \sum_{\nu} \int d\boldsymbol{r} \rho_\nu^\infty G_{s\nu}(\boldsymbol{r}) \ln\big(G_{s\nu}(\boldsymbol{r})\big) \\
- \frac{1}{2}\sum_{\nu}\sum_{\nu'} \rho_\nu^\infty \rho_{\nu'}^\infty \int\int d\boldsymbol{r}\, d\boldsymbol{r'} G_{s\nu}(\boldsymbol{r}) \Big[G_{s\nu'}(\boldsymbol{r'}) \left(g_{\nu,\nu'}^{inh}(\boldsymbol{r},\boldsymbol{r'}) \ln\left(g_{\nu,\nu'}^{inh}(\boldsymbol{r},\boldsymbol{r'})\right)\right. \\
\left. - g_{\nu,\nu'}^{inh}(\boldsymbol{r},\boldsymbol{r'}) + 1\right) - \left(g_{\nu\nu'}^0(\boldsymbol{r},\boldsymbol{r'}) \ln g_{\nu\nu'}^0(\boldsymbol{r},\boldsymbol{r'}) - g_{\nu\nu'}^0(\boldsymbol{r},\boldsymbol{r'}) + 1\right)\Big] \\
- \frac{1}{2}\sum_{\nu}\sum_{\nu'} \rho_\nu^0 \rho_{\nu'}^0 V_{ex,\nu} \int g_{\nu\nu'}^0(\boldsymbol{r'}) \ln g_{\nu\nu'}^0(\boldsymbol{r'}) - g_{\nu\nu'}^0(\boldsymbol{r'}) + 1\, d\boldsymbol{r'}
\end{aligned} \tag{33}
$$

As discussed above, $g_{\nu,\nu'}^{inh}$ does not become equal to $g_{\nu,\nu'}^0$ in the bulk, due to the different definition of the density. However, $G_{s\nu}$ becomes 1 in the bulk. This allows us to split the integrals into a local (close to the solute) and a distant region.

For the energy:

$$\frac{1}{2}\sum_{\nu}\sum_{\nu'}\rho_\nu^\infty\rho_{\nu'}^\infty\int\int d\boldsymbol{r}\, d\boldsymbol{r}' G_{s\nu}(\boldsymbol{r})\left[G_{s\nu'}(\boldsymbol{r}')g_{\nu,\nu'}^{inh}(\boldsymbol{r},\boldsymbol{r}') - g_{\nu\nu'}^{0}(\boldsymbol{r},\boldsymbol{r}')\right]u_{\nu\nu'}(\boldsymbol{r},\boldsymbol{r}')$$
$$= \frac{1}{2}\sum_{\nu}\sum_{\nu'}\rho_\nu^\infty\rho_{\nu'}^\infty\int_{local} d\boldsymbol{r}\, d\boldsymbol{r}' G_{s\nu}(\boldsymbol{r})\left[G_{s\nu'}(\boldsymbol{r}')g_{\nu,\nu'}^{inh}(\boldsymbol{r},\boldsymbol{r}') - g_{\nu\nu'}^{0}(\boldsymbol{r},\boldsymbol{r}')\right]u_{\nu\nu'}(\boldsymbol{r},\boldsymbol{r}') + \frac{1}{2}\sum_{\nu}\sum_{\nu'}\rho_\nu^\infty\rho_{\nu'}^\infty V\int_V d\boldsymbol{r}'\left[g_{\nu,\nu'}^{inh}(\boldsymbol{r}') - g_{\nu\nu'}^{0}(\boldsymbol{r}')\right]u_{\nu\nu'}(\boldsymbol{r}') \tag{34}$$

For the entropy:

$$\frac{1}{2}\sum_{\nu}\sum_{\nu'}\rho_\nu^\infty\rho_{\nu'}^\infty\int\int d\boldsymbol{r}\, d\boldsymbol{r}' G_{s\nu}(\boldsymbol{r})\Big[G_{s\nu'}(\boldsymbol{r}')\left(g_{\nu,\nu'}^{inh}(\boldsymbol{r},\boldsymbol{r}')\, ln\left(g_{\nu,\nu'}^{inh}(\boldsymbol{r},\boldsymbol{r}')\right) - g_{\nu,\nu'}^{inh}(\boldsymbol{r},\boldsymbol{r}') + 1\right) - \left(g_{\nu\nu'}^{0}(\boldsymbol{r},\boldsymbol{r}')\, ln\, g_{\nu\nu'}^{0}(\boldsymbol{r},\boldsymbol{r}') - g_{\nu\nu'}^{0}(\boldsymbol{r},\boldsymbol{r}') + 1\right)\Big]$$
$$= \frac{1}{2}\sum_{\nu}\sum_{\nu'}\rho_\nu^\infty\rho_{\nu'}^\infty\int_{local} d\boldsymbol{r}\, d\boldsymbol{r}' G_{s\nu}(\boldsymbol{r})\Big[G_{s\nu'}(\boldsymbol{r}')\left(g_{\nu,\nu'}^{inh}(\boldsymbol{r},\boldsymbol{r}')\, ln\left(g_{\nu,\nu'}^{inh}(\boldsymbol{r},\boldsymbol{r}')\right) - g_{\nu,\nu'}^{inh}(\boldsymbol{r},\boldsymbol{r}') + 1\right) - \left(g_{\nu\nu'}^{0}(\boldsymbol{r},\boldsymbol{r}')\, ln\, g_{\nu\nu'}^{0}(\boldsymbol{r},\boldsymbol{r}') - g_{\nu\nu'}^{0}(\boldsymbol{r},\boldsymbol{r}') + 1\right)\Big] + \frac{1}{2}\sum_{\nu}\sum_{\nu'}\rho_\nu^\infty\rho_{\nu'}^\infty V\int_V d\boldsymbol{r}'\left[\left(g_{\nu,\nu'}^{inh}(\boldsymbol{r}')\, ln\, g_{\nu,\nu'}^{inh}(\boldsymbol{r}') - g_{\nu,\nu'}^{inh}(\boldsymbol{r}') + 1\right) - \left(g_{\nu\nu'}^{0}(\boldsymbol{r}')\, ln\, g_{\nu\nu'}^{0}(\boldsymbol{r}') - g_{\nu\nu'}^{0}(\boldsymbol{r}') + 1\right)\right] \tag{35}$$

In the distant region, the difference between g(inh) and g(0) is mainly due to the density difference. Therefore, we approximate the difference between the $g\ln g - g + 1$ terms in terms of the density-difference, which in turn can be expressed as $\Delta\rho_{\nu'} = \rho_{\nu'}V_{ex,\nu'}/V$. For the energy:

$$\frac{1}{2}\sum_{\nu}\sum_{\nu'}\rho_\nu^\infty\rho_{\nu'}^\infty V\int_V d\boldsymbol{r}'\left[g_{\nu,\nu'}^{inh}(\boldsymbol{r}') - g_{\nu\nu'}^{0}(\boldsymbol{r}')\right]u_{\nu\nu'}(\boldsymbol{r}')$$
$$= \frac{1}{2}\sum_{\nu}\sum_{\nu'}\rho_\nu^\infty\rho_{\nu'}^\infty V\frac{\partial}{\partial\rho_{\nu'}}\int_V d\boldsymbol{r}'\left[g_{\nu\nu'}^{0}(\boldsymbol{r}')u_{\nu\nu'}(\boldsymbol{r}')\right]\Delta\rho_{\nu'}$$
$$= \frac{1}{2}\sum_{\nu}\sum_{\nu'}\rho_\nu^\infty{\rho_{\nu'}^\infty}^2\frac{\partial}{\partial\rho_{\nu'}}\int_V d\boldsymbol{r}'\left[g_{\nu\nu'}^{0}(\boldsymbol{r}')u_{\nu\nu'}(\boldsymbol{r}')\right]V_{ex,\nu'}$$
$$\approx \frac{1}{2}\sum_{\nu}\sum_{\nu'}\rho_\nu^0{\rho_{\nu'}^0}^2\frac{\partial}{\partial\rho_{\nu'}}\int_V d\boldsymbol{r}'\left[g_{\nu\nu'}^{0}(\boldsymbol{r}')u_{\nu\nu'}(\boldsymbol{r}')\right]V_{ex,\nu'} \tag{36}$$

For the entropy:

$$
\begin{aligned}
&\frac{1}{2}\sum_{\nu}\sum_{\nu'}\rho_{\nu}^{\infty}\rho_{\nu'}^{\infty}V\int_{V}d\boldsymbol{r}'\left[\left(g_{\nu,\nu'}^{inh}(\boldsymbol{r}')\,ln\,g_{\nu,\nu'}^{inh}(\boldsymbol{r}')-g_{\nu,\nu'}^{inh}(\boldsymbol{r}')+1\right)\right.\\
&\qquad\left.-\left(g_{\nu\nu'}^{0}(\boldsymbol{r}')\,ln\,g_{\nu\nu'}^{0}(\boldsymbol{r}')-\ g_{\nu\nu'}^{0}(\boldsymbol{r}')+1\right)\right]\\
&\qquad=\frac{1}{2}\sum_{\nu}\sum_{\nu'}\rho_{\nu}^{\infty}\rho_{\nu'}^{\infty}V\int_{V}d\boldsymbol{r}'\frac{\partial}{\partial\rho_{\nu'}}\left[g_{\nu,\nu'}^{0}(\boldsymbol{r}')\,ln\,g_{\nu,\nu'}^{0}(\boldsymbol{r}')-g_{\nu,\nu'}^{0}(\boldsymbol{r}')+1\right]\Delta\rho_{\nu'}\\
&\qquad=\frac{1}{2}\sum_{\nu}\sum_{\nu'}\rho_{\nu}^{\infty}\rho_{\nu'}^{\infty\,2}\int_{V}d\boldsymbol{r}'\frac{\partial}{\partial\rho_{\nu'}}\left[g_{\nu,\nu'}^{0}(\boldsymbol{r}')\,ln\,g_{\nu,\nu'}^{0}(\boldsymbol{r}')-g_{\nu,\nu'}^{0}(\boldsymbol{r}')+1\right]V_{ex,\nu'}\\
&\qquad\approx\frac{1}{2}\sum_{\nu}\sum_{\nu'}\rho_{\nu}^{0}{\rho_{\nu'}^{0}}^{2}\int_{V}d\boldsymbol{r}'\frac{\partial}{\partial\rho_{\nu'}}\left[g_{\nu,\nu'}^{0}(\boldsymbol{r}')\,ln\,g_{\nu,\nu'}^{0}(\boldsymbol{r}')-g_{\nu,\nu'}^{0}(\boldsymbol{r}')+1\right]V_{ex,\nu'}
\end{aligned}
\tag{37}
$$

In the following, we drop the index V on the integral. The V_{ex}-dependent terms can be combined using the product rule for derivatives with $\frac{\partial}{\partial\rho_{\nu'}}\rho_{\nu'}\times\int_V g_{\nu\nu'}^{0}(\boldsymbol{r}')u_{\nu\nu'}(\boldsymbol{r}')\mathrm{d}\boldsymbol{r}'$. Inserting those results into the partial molar energy and entropy gives:
For the energy:

$$
\begin{aligned}
\bar{e}_{s}^{\infty}&=\frac{3}{2}kT+\sum_{\nu}\rho_{\nu}^{\infty}\int d\boldsymbol{r}\,G_{s\nu}(\boldsymbol{r})u_{s\nu}(\boldsymbol{r})\\
&\quad+\frac{1}{2}\sum_{\nu}\sum_{\nu'}\rho_{\nu}^{\infty}\rho_{\nu'}^{\infty}\int_{local}d\boldsymbol{r}\,d\boldsymbol{r}'G_{s\nu}(\boldsymbol{r})\left[G_{s\nu'}(\boldsymbol{r}')g_{\nu,\nu'}^{inh}(\boldsymbol{r},\boldsymbol{r}')\right.\\
&\quad\left.-g_{\nu\nu'}^{0}(\boldsymbol{r},\boldsymbol{r}')\right]u_{\nu\nu'}(\boldsymbol{r},\boldsymbol{r}')+\frac{1}{2}\sum_{\nu}\sum_{\nu'}\rho_{\nu}^{0}{\rho_{\nu'}^{0}}^{2}V_{ex,\nu'}\frac{\partial}{\partial\rho_{\nu'}}\int g_{\nu\nu'}^{0}(\boldsymbol{r}')u_{\nu\nu'}(\boldsymbol{r}')d\boldsymbol{r}'\\
&\quad+\frac{1}{2}\sum_{\nu}\sum_{\nu'}\rho_{\nu}^{0}\rho_{\nu'}^{0}V_{ex,\nu'}\int g_{\nu\nu'}^{0}(\boldsymbol{r}')u_{\nu\nu'}(\boldsymbol{r}')d\boldsymbol{r}'\\
&=\frac{3}{2}kT+\sum_{\nu}\rho_{\nu}^{\infty}\int d\boldsymbol{r}\,G_{s\nu}(\boldsymbol{r})u_{s\nu}(\boldsymbol{r})\\
&\quad+\frac{1}{2}\sum_{\nu}\sum_{\nu'}\rho_{\nu}^{\infty}\rho_{\nu'}^{\infty}\int_{local}d\boldsymbol{r}\,d\boldsymbol{r}'G_{s\nu}(\boldsymbol{r})\left[G_{s\nu'}(\boldsymbol{r}')g_{\nu,\nu'}^{inh}(\boldsymbol{r},\boldsymbol{r}')\right.\\
&\quad\left.-g_{\nu\nu'}^{0}(\boldsymbol{r},\boldsymbol{r}')\right]u_{\nu\nu'}(\boldsymbol{r},\boldsymbol{r}')\\
&\quad+\frac{1}{2}\sum_{\nu}\sum_{\nu'}\rho_{\nu}^{0}\rho_{\nu'}^{0}V_{ex,\nu'}\frac{\partial}{\partial\rho_{\nu'}}\left[\rho_{\nu'}^{0}\int g_{\nu\nu'}^{0}(\boldsymbol{r}')u_{\nu\nu'}(\boldsymbol{r}')d\boldsymbol{r}'\right]
\end{aligned}
\tag{38}
$$

For the entropy:

$$\frac{\bar{s}^{\infty}}{k} = \frac{3}{2} - \frac{ln(\rho_s \lambda_s^3)}{k} - \sum_{\nu} \rho_\nu V_{ex,\nu} - \sum_{\nu} \int d\boldsymbol{r} \rho_\nu^\infty G_{s\nu}(\boldsymbol{r})\, ln\big(G_{s\nu}(\boldsymbol{r})\big)$$
$$- \frac{1}{2}\sum_{\nu}\sum_{\nu'} \rho_\nu^\infty \rho_{\nu'}^\infty \int_{local} d\boldsymbol{r}\, d\boldsymbol{r}' G_{s\nu}(\boldsymbol{r}) \Big[G_{s\nu'}(\boldsymbol{r}') \ \Big(g_{\nu,\nu'}^{inh}(\boldsymbol{r},\boldsymbol{r}')\, ln\Big(g_{\nu,\nu'}^{inh}(\boldsymbol{r},\boldsymbol{r}')\Big)$$
$$- g_{\nu,\nu'}^{inh}(\boldsymbol{r},\boldsymbol{r}') + 1\Big) - \big(g_{\nu\nu'}^{0}(\boldsymbol{r},\boldsymbol{r}')\, ln\, g_{\nu\nu'}^{0}(\boldsymbol{r},\boldsymbol{r}') - \ g_{\nu\nu'}^{0}(\boldsymbol{r},\boldsymbol{r}') + 1\big)\Big]$$
$$- \frac{1}{2}\sum_{\nu}\sum_{\nu'} \rho_\nu^0 {\rho_{\nu'}^0}^2 V_{ex,\nu'} \frac{\partial}{\partial \rho_{\nu'}} \int_V \big[g_{\nu,\nu'}^0(\boldsymbol{r}')\, ln\, g_{\nu,\nu'}^0(\boldsymbol{r}') - g_{\nu,\nu'}^0(\boldsymbol{r}') + 1\big] d\boldsymbol{r}'$$
$$- \frac{1}{2}\sum_{\nu}\sum_{\nu'} \rho_\nu^0 \rho_{\nu'}^0 V_{ex,\nu'} \int g_{\nu\nu'}^0(\boldsymbol{r}')\, ln\, g_{\nu\nu'}^0(\boldsymbol{r}') - \ g_{\nu\nu'}^0(\boldsymbol{r}') + 1\, d\boldsymbol{r}' \qquad (39)$$
$$= \frac{3}{2} - \frac{ln(\rho_s \lambda_s^3)}{k} - \sum_{\nu} \rho_\nu V_{ex,\nu} - \sum_{\nu} \int d\boldsymbol{r} \rho_\nu^\infty G_{s\nu}(\boldsymbol{r})\, ln\big(G_{s\nu}(\boldsymbol{r})\big)$$
$$- \frac{1}{2}\sum_{\nu}\sum_{\nu'} \rho_\nu^\infty \rho_{\nu'}^\infty \int_{local} d\boldsymbol{r}\, d\boldsymbol{r}' G_{s\nu}(\boldsymbol{r}) \Big[G_{s\nu'}(\boldsymbol{r}') \ \Big(g_{\nu,\nu'}^{inh}(\boldsymbol{r},\boldsymbol{r}')\, ln\Big(g_{\nu,\nu'}^{inh}(\boldsymbol{r},\boldsymbol{r}')\Big)$$
$$- g_{\nu,\nu'}^{inh}(\boldsymbol{r},\boldsymbol{r}') + 1\Big) - \big(g_{\nu\nu'}^{0}(\boldsymbol{r},\boldsymbol{r}')\, ln\, g_{\nu\nu'}^{0}(\boldsymbol{r},\boldsymbol{r}') - \ g_{\nu\nu'}^{0}(\boldsymbol{r},\boldsymbol{r}') + 1\big)\Big]$$
$$- \frac{1}{2}\sum_{\nu}\sum_{\nu'} \rho_\nu^0 \rho_{\nu'}^0 V_{ex,\nu'} \frac{\partial}{\partial \rho_{\nu'}} \rho_{\nu'}^0 \int_V \big[g_{\nu,\nu'}^0(\boldsymbol{r}')\, ln\, g_{\nu,\nu'}^0(\boldsymbol{r}') - g_{\nu,\nu'}^0(\boldsymbol{r}') + 1\big] d\boldsymbol{r}'$$

As in Lazaridis' derivation, we split the energy and entropy into "local" and "liberation" terms. Lazaridis further replaces the remaining ρ^0-terms by ρ^∞, This simplification, together with assuming a single solvent and $V_{ex} = -\kappa k T$, makes our results equal to Lazaridis original equations:

$$\Delta E^* = \sum_{\nu} \rho_\nu^\infty \int d\boldsymbol{r}\ G_{s\nu}(\boldsymbol{r}) u_{s\nu}(\boldsymbol{r})$$
$$+ \frac{1}{2}\sum_{\nu}\sum_{\nu'} \rho_\nu^\infty \rho_{\nu'}^\infty \int_{local} G_{s\nu}(\boldsymbol{r}) \big[G_{s\nu'}(\boldsymbol{r}') g_{\nu,\nu'}^{inh}(\boldsymbol{r},\boldsymbol{r}') \qquad (40)$$
$$- g_{\nu\nu'}^0(\boldsymbol{r},\boldsymbol{r}')\big] u_{\nu\nu'}(\boldsymbol{r},\boldsymbol{r}') d\boldsymbol{r}\, d\boldsymbol{r}'$$

$$\frac{\Delta S^*}{k} = - \sum_{\nu} \int d\boldsymbol{r} \rho_\nu^\infty G_{s\nu}(\boldsymbol{r})\, ln\big(G_{s\nu}(\boldsymbol{r})\big)$$
$$- \frac{1}{2}\sum_{\nu}\sum_{\nu'} \rho_\nu^\infty \rho_{\nu'}^\infty \int_{local} d\boldsymbol{r}\, d\boldsymbol{r}' G_{s\nu}(\boldsymbol{r}) \Big[G_{s\nu'}(\boldsymbol{r}') \ \Big(g_{\nu,\nu'}^{inh}(\boldsymbol{r},\boldsymbol{r}')\, ln\Big(g_{\nu,\nu'}^{inh}(\boldsymbol{r},\boldsymbol{r}')\Big) \qquad (41)$$
$$- g_{\nu,\nu'}^{inh}(\boldsymbol{r},\boldsymbol{r}') + 1\Big) - \big(g_{\nu\nu'}^{0}(\boldsymbol{r},\boldsymbol{r}')\, ln\, g_{\nu\nu'}^{0}(\boldsymbol{r},\boldsymbol{r}') - \ g_{\nu\nu'}^{0}(\boldsymbol{r},\boldsymbol{r}') + 1\big)\Big]$$

$$e^{lib} = \frac{3}{2} kT + \frac{1}{2}\sum_{\nu}\sum_{\nu'} \rho_\nu^0 \rho_{\nu'}^0 V_{ex,\nu'} \frac{\partial}{\partial \rho_{\nu'}} \Big[\rho_{\nu'}^0 \int g_{\nu\nu'}^0(\boldsymbol{r}') u_{\nu\nu'}(\boldsymbol{r}') d\boldsymbol{r}'\Big] \qquad (42)$$

$$\frac{s_{lib}}{k} = \frac{3}{2} - \frac{ln(\rho_s \lambda_s^3)}{k} - \sum_{\nu} \rho_\nu V_{ex,\nu'} - \frac{1}{2}\sum_{\nu}\sum_{\nu'} \rho_\nu^0 \rho_{\nu'}^0 V_{ex,\nu'} \frac{\partial}{\partial \rho_{\nu'}} \rho_{\nu'}^0 \int_V \left[g_{\nu,\nu'}^0(\boldsymbol{r}') \, ln \, g_{\nu,\nu'}^0(\boldsymbol{r}') - g_{\nu,\nu'}^0(\boldsymbol{r}') + 1\right] d\boldsymbol{r}' \quad (43)$$

1. Lazaridis, T., *Inhomogeneous fluid approach to solvation thermodynamics. 1. Theory.* Journal of Physical Chemistry B, 1998. **102**(18): p. 3531-3541.
2. Morita, T. and K. Hiroike, A New Approach to the Theory of Classical Fluids .3. General Treatment of Classical Systems. Progress of Theoretical Physics, 1961. **25**(4): p. 537-578.
3. Isihara, A., *Statistical physics*. 1971, New York,: Academic Press. xv, 438 p.
